# Nonuniform temperature and light relationships of moss‐associated nitrogen fixation across climates

**DOI:** 10.1111/nph.71472

**Published:** 2026-08-03

**Authors:** Yunyao Ma, Kathrin Rousk

**Affiliations:** ^1^ Department of Biology University of Copenhagen Copenhagen 2100 Denmark; ^2^ Center for Volatile Interactions University of Copenhagen Copenhagen 2100 Denmark

**Keywords:** climate‐response curves, moss–cyanobacteria association, nitrogen fixation, nitrogen fixation modeling, nitrogenase, physiological response traits of N fixation

## Abstract

- Nitrogen (N) fixation by moss‐associated cyanobacteria is a key N source in terrestrial ecosystems and thereby a critical limiting factor for ecosystem productivity. However, the responses of this process to variation in key abiotic factors are unknown. In particular, the lack of knowledge on how these responses vary across climates limits our ability to estimate and predict the global importance of this key ecosystem function.
- We established N fixation response curves in dominant mosses across five ecosystems with different climates, from arctic to tropical forests, to light (0–1500 μmol m^−2^ s^−1^), moisture (0–100% moss moisture), and temperature (< 10–40°C), and derived key physiological traits from these.
- Relationships between N fixation and both light and temperature varied across climatically contrasting ecosystems, with light optima of *c.* 500–800 μmol m^−2^ s^−1^ and temperature optima of *c.* 28–33°C. By contrast, the amount of water required to sustain N fixation was similar across ecosystems, regardless of local humidity.
- Our results highlight that microbial functional responses are not uniform across climates. The here quantified relationships between N fixation and key abiotic factors across climatically contrasting ecosystems provide the foundation for integrating moss‐associated N fixation into vegetation models.

## Graphical abstract

Light, moisture, and temperature response of nitrogen fixation in moss–cyanobacteria associations across five climatically contrasting ecosystems. Responses of nitrogen fixation in moss–cyanobacteria associations to light (μmol photons m^−2^ s^−1^), moss relative water content (%), and temperature (°C) across five climatically contrasting ecosystems.

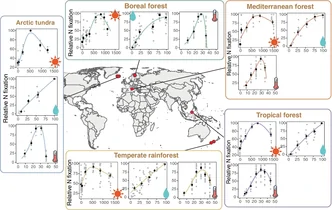

## Introduction

Nitrogen (N) is an essential nutrient that limits plant productivity, particularly in the face of climate change that is expected to substantially increase plant N demand (Reich *et al*., 2006; Wang & Houlton, 2009; Kou *et al*., 2020). Biological N fixation by moss–cyanobacteria associations is an important N source, as it can provide 50% of ‘new’ N in terrestrial ecosystems, especially in moss‐rich boreal forests and arctic tundra (DeLuca *et al*., 2002; Ramm *et al*., 2022). It also contributes to meeting the increased plant‐N demand under warming in permafrost ecosystems, which partially alleviates N limitation and ultimately sustains plant CO_2_ uptake (Zhou *et al*., 2026). However, despite growing interest in understanding global N fixation rates by moss–cyanobacteria associations (Elbert *et al*., 2012; Lindo *et al*., 2013; Slate *et al*., 2024) and their controls, such as light, water, and temperature (Rousk, 2022), studies on moss‐associated N fixation remain relatively limited compared to those on for example leguminous N fixation (Rousk & Villarreal, 2025). In particular, our knowledge of the relationship between N fixation in mosses and abiotic drivers across global climates is still limited: Most studies have compared N fixation rates by moss–cyanobacteria associations at a maximum 2–3 levels of temperature, light, or moisture (Gundale *et al*., 2012a; Rousk *et al*., 2017; Permin *et al*., 2022), and focused on a certain abiotic factor in selected ecosystems (Fan *et al*., 2022; Alvarenga *et al*., 2024a). This is insufficient to cover the entire range of variations found in nature and thus cannot reveal the full relationship between N fixation and these abiotic factors, and how they differ across climates. As a result, a comprehensive, quantitative integration of N fixation with the entire range of abiotic drivers across climates is still lacking. This knowledge is not only essential for understanding the physiological properties and ecological strategies of cyanobacteria living on plant leaves (e.g. mosses) across diverse environments but also clearly needed for predicting spatiotemporal variation in N fixation in the long‐term using process‐based vegetation models.

Biological N fixation is an enzymatic process with a temperature optimum initially proposed to be *c*. 25°C (Houlton *et al*., 2008). However, recent data have indicated that this value can be higher than 30°C and can vary between ecosystems (Bytnerowicz *et al*., 2022). In mosses, the temperature optimum of associated N fixation ranges between 20 and 32°C across temperate, boreal, and arctic climates (Zielke *et al*., 2002; Jean *et al*., 2012; Gundale *et al*., 2012a; Rousk *et al*., 2017). Additionally, cyanobacterial N fixation activity on mosses under extreme warm conditions exhibits thermal adaptation to local temperature regimes in a temperate habitat (Jean *et al*., 2012). However, warm‐climate habitats such as tropical and mediterranean regions remain understudied, and quantitative assessments of optimum temperatures and thermal limits for N fixation across climates are still lacking. Thereby, quantifying the response of N fixation to the full range of temperature across climates is needed for predicting large‐scale N fixation patterns under current and future climate warming scenarios.

N fixation is a highly energy‐demanding process and is therefore expected to increase with light intensity, as light is critical for supplying energy and supporting protein synthesis via photosynthesis (Vitousek *et al*., 2002; Adams & Duggan, 2008). On the other hand, the positive impact of increasing light intensity might saturate or even become inhibiting at high light levels due to potential photodegradation of the moss host or its cyanobionts (Hájek *et al*., 2009). Under high light and temperatures, moss moisture content decreases, with negative impacts on associated N fixation rates (Rousk, 2022). Indeed, a range of studies on moss‐associated N fixation have suggested that moisture is a key limiting factor of N fixation (Zielke *et al*., 2002; Stewart *et al*., 2011; Rousk *et al*., 2015; Li *et al*., 2018). Yet, excessive water content may suppress N fixation by inhibiting N_2_ diffusion and thereby reducing its availability for N fixation, analogous to CO_2_ limitation in photosynthesis (Lange & Tenhunen, 1981; Lange *et al*., 1993). While studies have examined the effects of a few light and water levels on N fixation, the response patterns of moss‐cyanobacteria N fixation to these factors remain unclear, and it is unknown whether these responses vary across climates.

Cyanobacterial N fixation is commonly linked to the biomass of colonizing cyanobacteria (DeLuca *et al*., 2007; Arróniz‐Crespo *et al*., 2014; Renaudin *et al*., 2021), which is similarly affected by environmental factors such as temperature and moisture (Renaudin *et al*., 2022). However, these environmental factors may impact N fixation and cyanobacterial biomass differently, also depending on the climate of origin, thereby affecting N fixation efficiency (i.e. the N fixation potential per unit cyanobacterial biomass). Evidence from studies on leguminous N fixation suggests that N fixation efficiency can increase under environmental stress, such as phosphorus limitation (Ribet & Drevon, 1996; McCulloch & Porder, 2020). Nevertheless, for moss‐associated N fixation, it remains largely unknown whether N fixation efficiency differs across climates.

In this study, we addressed the following two questions: (1) What are the light, water, and temperature responses of N fixation, and N fixation efficiency in moss–cyanobacteria associations from ecosystems with different climates? (2) Which mechanisms underlie the variation in moss–cyanobacteria N fixation responses across climates? We hypothesized that: (H1) N fixation responses vary among climatically contrasting ecosystems, reflecting the local climate conditions of the sampled habitats. Specifically, (1) mosses from low‐light environments have higher associated N fixation rates under low light compared to mosses from high‐light environments; (2) N fixation associated with mosses from dry climates (i.e. mediterranean) require less water to be active and peak at lower water content compared to N fixation in mosses from wet climates (i.e. tropics); and (3) the optimum temperature for N fixation is higher in warmer climates (tropical and mediterranean) compared to cooler climates (arctic and boreal); (H2) N fixation efficiency is higher in mosses from harsher environments (mediterranean with frequent drought events vs constantly wet tropics), reflecting locally shaped physiology to short periods of activity. To address these hypotheses, we measured N fixation across gradients of light (6 levels from 0 to 1500 μmol m^−2^ s^−1^), relative moss‐water content (5 levels from dry to 100% water saturation), and temperature (5 levels from < 10–40°C) in dominant moss species collected from five ecosystems with different climates (arctic, boreal, temperate, mediterranean, and tropical). We then quantified key response traits of N fixation, such as optimum temperature, optimal light intensity, and the minimum water content required for activation. By comparing these response traits and N fixation efficiency, we can assess how moss‐associated N fixation physiology varies across climates.

## Materials and Methods

### Sample collection

**Fig. 1:**
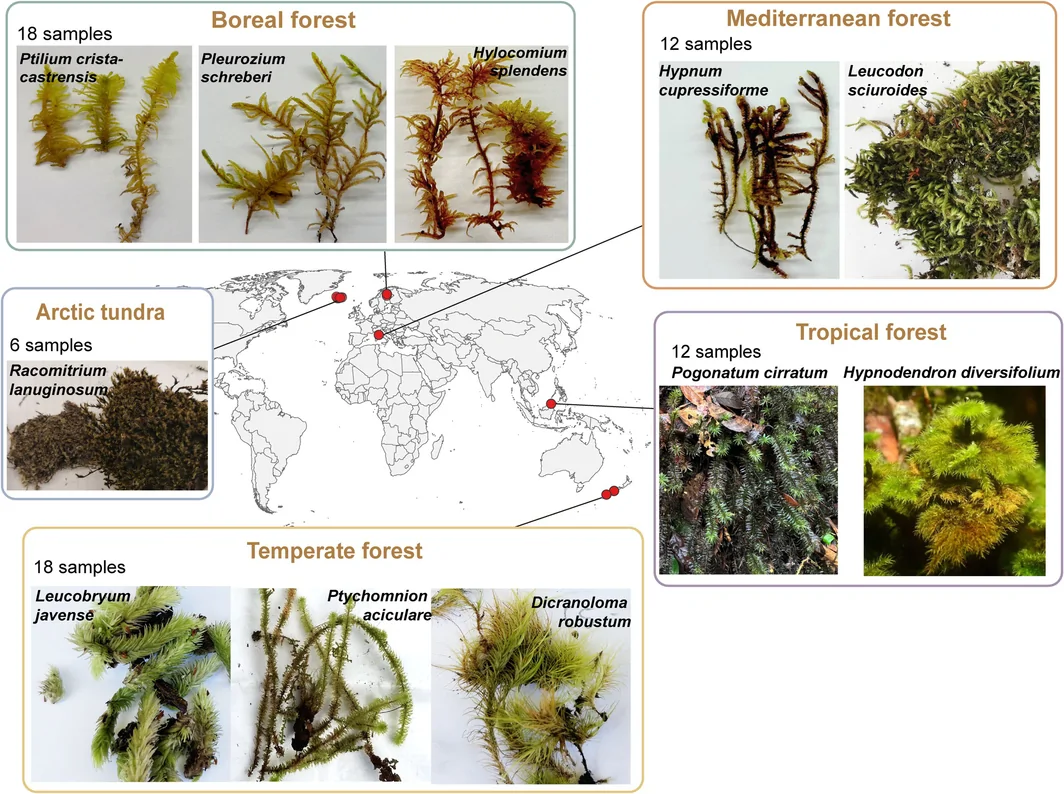
Geographic distribution of moss sampling sites and collected species within five ecosystems spanning contrasting climates: arctic tundra, boreal forest, temperate rainforest, mediterranean forest, and tropical forest. Photographs show the dominant mosses collected (*n* = 6) and assessed in this study.

Samples containing one to three dominant moss species were collected from sites within five unmanaged ecosystems spanning different climates: arctic tundra, boreal forest, temperate rainforest, mediterranean forest, and tropical forest (Fig. 1; see Supporting Information Table S1 for details). Dominant moss species were selected based on prior knowledge and regional distribution records in the Global Biodiversity Information Facility (GBIF; https://www.gbif.org/), and visually confirmed at the respective sampling sites during field collection. Sampling was conducted at peak productivity (spring–summer) in the sites with seasons (arctic, boreal, temperate, mediterranean sites) and in the wet season in the tropical site. Within each site, shoots of these moss species were collected from replicate plots (*n* = 6) with at least 5 m apart.

Samples were placed in transparent ziplock bags to maintain their original moisture status (dry at boreal and mediterranean forests, moist at the other sites) and transported dark and cool to the University of Copenhagen, Denmark where they were kept at 4°C in darkness. Samples from warm habitats (tropical and mediterranean forests) were processed within 2 wk after collection. Samples from the cold habitats (arctic and boreal) were stored for 2 months before processing, while samples from the temperate forests were stored for *c*. 7 months due to practical constraints. N‐fixing microbes from all sites are expected to remain largely inactive under these storage conditions (Puzorjov & McCormick, 2020; see also Notes S1.1). The temperate samples, which had the longest storage duration, showed no significant difference in N fixation activity after storage compared with freshly measured samples collected directly from the field (*P* > 0.05). The storage conditions are therefore unlikely to have substantially altered the physiological response patterns to abiotic drivers (see Notes S1.1).

### N‐fixation response measurements

One week before the experiments, moss shoots were cleaned of any soil or bark attached to them. Then, moss shoots were submerged in distilled water for 10 min to completely saturate them. Randomly selected shoots were then shaken slightly to remove excess water and placed in a sieve for 2 min to allow excess water to drip off. Any remaining excess water was carefully removed by blotting paper. The samples were then weighed to obtain the maximum water content (WCmax). We then placed the samples in 20‐ml glass vials and kept them in a growth chamber (Aralab FitoClima 1.200 PLH, Rio de Mouro, Portugal) for 1 wk, with a 12 h : 12 h, light : dark cycle, a photosynthetically active radiation (PAR) of 150 μmol m^−2^ s^−1^, a temperature of 20°C in the light and 17°C in the dark, and relative humidity of 80%. Samples were gently sprayed with distilled water once a day to prevent drying out. This procedure allowed all samples to reactivate after storage and ensured similar conditions for all moss samples before the measurements (see detailed explanation in the Notes S1.1).

Subsequently, the first nitrogenase activity was measured under conditions of 50 μmol photons m^−2^ s^−1^, 25°C, and full water saturation, conditions that have been assumed to be near‐optimal for N fixation in forest understorey mosses (Gentili *et al*., 2005). Samples were then subjected to the experimental manipulations, which were conducted in the order of light, water, and temperature (will be discussed later). For the boreal mosses, we used three independent sample sets for the light, water, and temperature‐response curves, respectively. These initial measurements showed that N fixation activity was maintained after the different moss‐hydration levels. Based on this observation, and because one‐time drying–rewetting cycles are commonly experienced by mosses and associated bacteria under field conditions, we subsequently used one sample set for the light response measurements and one sample set for the water and temperature‐response curves. This design avoided using samples that had been exposed to potentially stressful high‐light or high‐temperature treatments in subsequent assays. Repeated measurements on the same sample per factor were used to characterize the response curves because N fixation activity can vary substantially within a species (Levinsen *et al*., 2025). Additional robustness tests indicated that the effects of repeated use of samples on response curves were negligible (Notes S1.2).

Nitrogenase activity was measured using the acetylene reduction assay (Schöllhorn & Burris, 1967). This method measures nitrogenase activity by quantifying the conversion of acetylene to ethylene catalyzed by the nitrogenase enzyme. The incubation was started by sealing the 20‐ml vials prepared in the previous step with rubber septa and replacing 2 ml of air (10% of the headspace) with 2 ml of acetylene gas using a syringe and needle. Samples were incubated in a growth chamber under the desired conditions for water or light‐response curves (will be discussed later) for 6 h, which is sufficiently short to produce detectable amounts of ethylene while keeping a balanced gas concentration inside the vials. However, we adjusted the incubation time for the temperature‐response curves to accurately capture the enzyme activity (Rousk *et al*., 2017) (will be discussed later). Furthermore, three control vials containing 10% acetylene gas only (no moss samples) were placed in the chamber alongside the samples, which were used to account for any residual ethylene present in the injected acetylene gas, and their values were subtracted from the samples' data. Ethylene production was measured using the Agilent 8890 Gas Chromatography System connected to an Agilent 7697A autosampler (Agilent, Santa Clara, USA). Throughout the measurements, we monitored the mosses to ensure that they remained visibly healthy to minimize potential carryover effects from previous measurement levels. Between measurements, samples were returned to the chamber at 20°C (17°C at night) with light of 150 μmol m^−2^ s^−1^, a 12 h : 12 h, light : dark cycle, and 80% relative humidity, and vials were opened to allow gas exchange to reduce potential effects of residual acetylene or ethylene from the previous incubation.

For the light‐response curves, samples were incubated at light intensities of 0, 50, 250, 500, 800, and 1300 μmol m^−2^ s^−1^ (in the boreal habitat, two of the three species (*Hylocomium splendens* (Hedw.) Schimp. and *Ptilium crista‐castrensis* (Hedw.) De Not) were additionally measured at *c*. 350 and 1000 μmol m^−2^ s^−1^) under optimal temperature (*c*. 25°C) and water conditions (at WCmax for all mosses, except 80% WCmax for boreal species) to ensure light was the only limiting factor. The actual light intensity was measured using a handheld quantum PAR meter (model MQ‐200; Apogee Instruments, Logan, UT, USA) and used to describe light levels. The darkness treatment was usually placed between high‐light treatments, for example in the sequence 250–0–500 μmol m^−2^ s^−1^, to ensure that samples had sufficient energy availability before and after dark incubation and that subsequent measurements were not strongly limited by the preceding darkness.

Water‐response curves were generated by stepwise adjusting the relative water content (RWC) of each sample to < 10, *c*. 25, *c*. 50, *c*. 75, and *c*. 100%, at optimal temperature (*c*. 25°C) and light (*c*. 580 μmol m^−2^ s^−1^ for most mosses; 320 μmol m^−2^ s^−1^ for temperate mosses) as determined from the light‐response curves. Specifically, 2 d before the measurements, samples were placed in open paper bags and left to dry gently until their weight had decreased to < 10% of their saturated weight and they appeared visibly dry. The samples were then sprayed with distilled water to reach the target water content and were usually maintained at this target moisture level for *c*. 2 h before incubation, allowing water to redistribute within the moss tissues. The RWC was calculated as (fresh weight – DW)/(WCmax – DW), where DW is the dry weight of samples that were determined after the measurements (will be discussed later). As we aimed to capture the immediate responses of N fixation, reflecting *in situ* physiology as closely as possible, the levels of light and RWC were increased daily for respective response curves.

The temperature‐response curves were achieved through incubating samples stepwise at 15, 8, 23, 28, 35, and 45°C, while keeping water and light levels constant and optimal (same as discussed previously) to isolate the effect of temperature. Measurements started at 15°C, a temperature close to conditions commonly experienced by all moss samples, followed by exposure to lower temperatures. After a recovery period of *c*. 1 d at 15°C, samples were then exposed to higher temperatures. This sequence was chosen to reduce potential cold‐ or heat‐shock effects. The actual temperature at the moss surface was measured using a digital thermometer placed in the same type of 20‐ml vial next to the experimental samples. These measured values were used to describe the temperature levels. Unlike the light and water responses, samples for the temperature‐response experiments were first acclimatized to the target incubation conditions in the growth chamber for 24 h before the incubations. The incubation time was adjusted to the exposure temperature: Incubation time was longer at low temperatures to ensure sufficient nitrogenase activity to be detected, and shortened at high temperatures to avoid underestimating N fixation rates due to high nitrogenase activity within a shorter time (Rousk *et al*., 2017; i.e., 2 h at 45 and 35°C; 4 h at 28°C; 6 h at 23°C; 10 h at 15°C; and 24 h at 8°C).

In total, 1068 measurements were conducted to establish all response curves. After completing all response measurements, samples were frozen at −20°C for 24 h and then freeze‐dried and weighed. Finally, N fixation rates were expressed as acetylene reduction per moss dry mass per incubation time (nmol g^−1^ h^−1^).

### Cyanobacterial phycocyanin content analysis

Cyanobacterial biomass in all moss samples used for the N fixation response measurements was estimated by measuring phycocyanin content, as phycocyanin is a cyanobacterial‐specific pigment (Renaudin *et al*., 2021), and cyanobacterial communities associated with mosses are often dominated by N‐fixing taxa (Holland‐Moritz *et al*., 2021). In short, the freeze‐dried samples were ground to a fine powder, and the phycocyanin content in the samples was extracted following a modified protocol based on Renaudin *et al*. (2021). Specifically, each sample was placed in a 15‐ml Falcon‐type tube and mixed with 10 ml of sterile sodium phosphate buffer (pH = 7). The samples were then vortexed and manually shaken before undergoing two freeze–thaw cycles (freezing at −20°C for at least 2 h followed by complete thawing at 25°C). Subsequently, the mixture was sonicated for 5 min and then centrifuged at 3739 ***g*** for 15 min. A total of 100 μl of each sample supernatant was transferred to a 96‐well microplate. The phycocyanin content was analyzed using a Synergy HT microplate reader (AH diagnostics) by measuring fluorescence with excitation at 590 nm and emission at 645 nm, and related to standards (C‐phycocyanin standard (catalog no.: 52468‐1MG‐F; Sigma‐Aldrich) with concentration from 0 to 250 μg l^−1^).

Based on these measurements, the N fixation efficiency was calculated as the N fixation capacity normalized to the phycocyanin content, where N fixation capacity was defined as the maximum N fixation rate (determined via acetylene reduction per moss dry mass per incubation time) across gradients of light, water, or temperature.

### Data analysis

#### Response functions for N fixation

The functions used to model the light, water, and temperature responses of N fixation were chosen based on the best fit to the empirical data. The light‐response curve was described best using a modified logistic function with a declining term to account for the inhibition of N fixation caused by high light stress (Hájek *et al*., 2009). This function is described as follows:

(Eqn 1) $$Y=\max\left(0,\left(\frac{y_{\max}}{\left(1+e^{-a\left(\text{light}-b\right)}\right)}\right)-c\times \text{light}\right)$$

where *Y* is the N fixation rate as a function of instantaneous measurement of light intensity (light; with *Y* having the same meanings in subsequent Eqs 2, 3); *y*_max_ is the maximum N fixation at the optimal light intensity; the parameters *a*, *b*, and *c* characterize the function: *a* determines the steepness of the increase of the curve, *b* represents the inflection point (the light level at which the response is half of the maximum), and *c* controls the rate of decline of the curve.

The water‐response curve for N fixation was described by a function that has been used for net photosynthesis (Nikolić *et al*., 2024):

(Eqn 2) $$Y=\max\left(0,\frac{\left(a^{2}\mathrm{WC}\right)}{\left({\mathrm{WC}}^{2}+\mathrm{ab}\right)}+c\right)$$

where WC is the RWC, the parameters *a*, *b* represent the position and width of the optimum water content, respectively, while c adjusts the height of the curve.

To model the temperature response of N fixation, we applied a modified beta function, which has been previously applied to model temperature responses of N fixation in woody N‐fixing plants and plant growth (Yan & Hunt, 1999; Bytnerowicz *et al*., 2022). This function captures a hump‐shaped curve with a skewed peak (i.e. asymmetry around the temperature optimum), and provided the best fit to the empirical data:

(Eqn 3) $$Y=\max\left(0,y_{\max}\left(\frac{a-T}{a-T_{\mathrm{opt}}}\right){\left(\frac{T-b}{T_{\mathrm{opt}}-b}\right)}^{\frac{T_{\mathrm{opt}}-b}{a-T_{\mathrm{opt}}}}\right)$$

where *T* is the temperature; *y*_max_ represents the maximum N fixation rate at the optimum temperature (*T*_opt_), *a*, and *b* are the maximum and minimum temperatures at which activity is 0, respectively.

We then fitted response curves to the measured N fixation data from all individual samples across species within each ecosystem and across different levels of light, RWC, and temperature, using these equations with a maximum‐likelihood estimation approach. The N fixation rates were standardized to the maximum rate for each sample within the corresponding response curve. This standardization allows robust comparisons of response patterns and derived response traits among species and ecosystems.

#### Key traits of N fixation responses to light, water, and temperature

To quantitatively assess the variation of N fixation responses to light, water, and temperature across ecosystems with different climates, we estimated key physiological traits and their confidence intervals (*CIs*) at 95, 85, and 75% from the fitted response curves. These traits have biological significance to describe the responses: for example, the optimal light intensity reflects how efficiently moss–cyanobacteria associations from different ecosystems with contrasting climates use light for N fixation. Additionally, the RWC required to activate N fixation activity indicates how efficiently moss–cyanobacteria associations can utilize water to initiate N fixation. Furthermore, the optimum temperature (Topt) and the normalized N fixation rates at low (10°C; N‐fixcold) and high temperatures (35°C; N‐fixwarm) reflect how moss‐associated N fixation in ecosystems with contrasting climates is adapted to local temperature conditions and how they perform under cold and warm conditions, respectively.

#### Climatic drivers of the variation of N fixation response traits

To further identify the key factors driving the observed significant variation in N fixation‐response traits across ecosystems spanning contrasting climates (i.e. Topt, N‐fixwarm, optimal light intensity; see Fig. 3 later), we calculated a set of macroclimate variables linked to light and temperature for sites within the five sampled ecosystems using the ERA5 reanalysis dataset (https://cds.climate.copernicus.eu/dataset/derived‐near‐surface‐meteorological‐variables), which offers hourly data at a spatial resolution of *c*. 30 km for the period 2010–2019. Although these data are too coarse to fully represent the site‐level microclimatic conditions experienced by the moss samples, which may be more directly relevant to physiological response traits, they provide a broad climatic context for the sampling sites and allow us to explore whether differences in large‐scale climatic regimes among sites may explain variation in N fixation traits. For temperature variables specifically, macroclimatic temperature can capture both the temporal variation and magnitude of moss‐surface microclimatic temperature reasonably well (see Fig. S1). Predictor variables for Topt included annual mean temperature, mean temperature in the month of sampling and the previous month, as well as temperature seasonality (calculated as the SD of monthly mean temperature). For N‐fixwarm, analogous variables were used, with the addition of maximum temperatures (i.e. mean temperature of the warmest month and maximum daily temperature in the sampling period). In addition, the metabolic activity of moss–cyanobacteria associations may also be a key factor modulating the effects of abiotic factors on N fixation response traits. For instance, mosses in the mediterranean habitat may not need to adapt to hot summers because they maintain inactive during dry periods. Thereby, we also included temperature variables during the active period, such as the mean annual temperature during the active period, for analyzing Topt and N‐fixwarm. The hourly active time fraction (RWC ≥ 10%) was estimated using the process‐based model LiBry driven by the retrieved hourly ERA5 climate data, which has been applied and validated in various plots in different biomes and globally to provide relatively accurate estimates of the water balance/activity of mosses (Porada *et al*., 2013, 2018; Ma *et al*., 2023). For the light‐response trait (optimal light intensity), predictors included several above‐canopy radiation variables: annual total radiation, maximum daily total radiation, these variables during the active period, total radiation in the sampling month, average daily hours with radiation > 100 W m^−2^. We also included leaf area index (LAI), averaged over 2000–2020 at *c*. 5.5‐km spatial resolution (H. Ma, unpublished data), as a proxy for general canopy structure at the sampling sites because most moss samples were collected from forest understorey habitats (see Table S1). Although the LAI data cannot directly reflect the fine‐scale conditions experienced by the sampled mosses, the extracted LAI pattern was consistent with our field observations that tropical and temperate forest sites have a more closed canopy than mediterranean and boreal forest sites.

To estimate the relative importance of climatic variables on the variation of N fixation response traits in different climate zones, we performed separate maximum‐likelihood fits for each of the sampled species to estimate their N fixation response traits (e.g. Topt) and averaged the values for species located within the same ERA5 grid cell. Subsequently, a partial least squares (PLS) regression model was applied, as it is particularly suitable given the high degree of multicollinearity among predictor variables in our dataset. Variable importance in projection (VIP) scores were then calculated using Eqn S1 in the supplement to quantify the contribution of each predictor variable in explaining the response variable (i.e. light/temperature‐response physiological traits) in the PLS model, with values > 1 considered significant contributors (Chong & Jun, 2005; Mehmood *et al*., 2012). We then examined the relationship between the temperature‐response traits (e.g. Topt) and their most relevant predictor variables using a linear regression model.

To further support the interpretation of the climatic‐driver analysis that identifies the sampling‐period temperature as important to drive the temperature response of N fixation across ecosystems with contrasting climates (see Fig. 4 later), we conducted an additional sampling in November in the mediterranean region, contrasting with the summer sampling. For this additional sampling, we collected two dominant moss species, *Leucodon sciuroides* (Hedw.) Schwägr and *Leptodon smithii* (Dicks. ex Hedw.) F.Weber & D.Mohr, from six replicate plots (≥5 m apart) in a holm oak forest in southeastern France (43.74° N, 6.34° E). These samples were transported, processed, and measured only for temperature‐response curves following the same protocol as described previously for the temperature‐response experiment. Briefly, these samples were incubated under optimal light conditions (*c*. 550 μmol m^−2^ s^−1^), full water saturation, and five temperature levels (8, 15, 23, 28, and 35°C). After the incubation experiments, samples were freeze‐dried and weighed to express the N fixation rate (Acetylene reduction) per moss dry mass (nmol g^−1^ h^−1^).

#### Statistical analysis

All statistical analyses were performed using R v.4.2.3. We used the ‘mle2’ function in the bbmle package (Bolker & R Development Core Team, 2023) in R to fit the response functions by maximum likelihood to the empirical data. To evaluate model performance across different sets of starting values, we used the Akaike information criterion corrected for small sample sizes (AICc), given that the ratio of data points to parameters was < 40 (Burnham & Anderson, 2004). The best set of starting values was selected based on the lowest AICc. Next, key N fixation response traits and their 95, 85, and 75% confidence intervals (CIs) were estimated based on the fitted curves using the population prediction interval method (Bolker, 2008): Model parameters were resampled 1000 times from a multivariate normal distribution derived from the fitted model using the ‘mvrnorm’ function from the Mass package (Venables & Ripley, 2002). For each resampled parameter set, the N fixation response traits were recalculated, and the corresponding quantiles of these estimates were used to compute the CIs. PLS models were performed using the ‘plsr’ function in pls package (Liland *et al*., 2024) to rank the relative importance of predictor variables for the Topt, N‐fixwarm, and optimal light intensity, with VIP scores manually calculated from Eqn S1 in the Methods S1. Furthermore, linear relationships between the most important predictor variables and N fixation response traits were assessed using ‘lm’ function from the stats package (R Core Team, 2023). To assess differences in N fixation efficiency and capacity, as well as phycocyanin content, among ecosystems with contrasting climates, we first evaluated whether the data, or their Box–Cox‐transformed values, met the assumptions of normality and homogeneity of variance. Box–Cox transformations were performed using the ‘boxcox’ function from the Mass package (Venables & Ripley, 2002) to identify the optimal transformation parameter (λ) for each variable. If the transformed data still violated ANOVA assumptions, we employed the nonparametric Kruskal–Wallis test instead. Specifically, we conducted one‐way ANOVA using the ‘aov’ function or the Kruskal–Wallis test using ‘kruskal.test’ function from the stats package (R Core Team, 2023). Where significant differences were detected, we performed *post hoc* pairwise comparisons using either Tukey's Honest Significant Difference test (‘TukeyHSD’ function in the stat package (R Core Team, 2023)) for parametric data, or the Dunn–Bonferroni *post hoc* test (‘dunn_test’ function from the rstatix package (Kassambara, 2023)) for nonparametric data. All figures were generated using the ‘ggplot’ function from the ggplot2 package (Wickham, 2016).

## Results

### Nitrogen fixation response patterns across ecosystems with contrasting climates

**Fig. 2:**
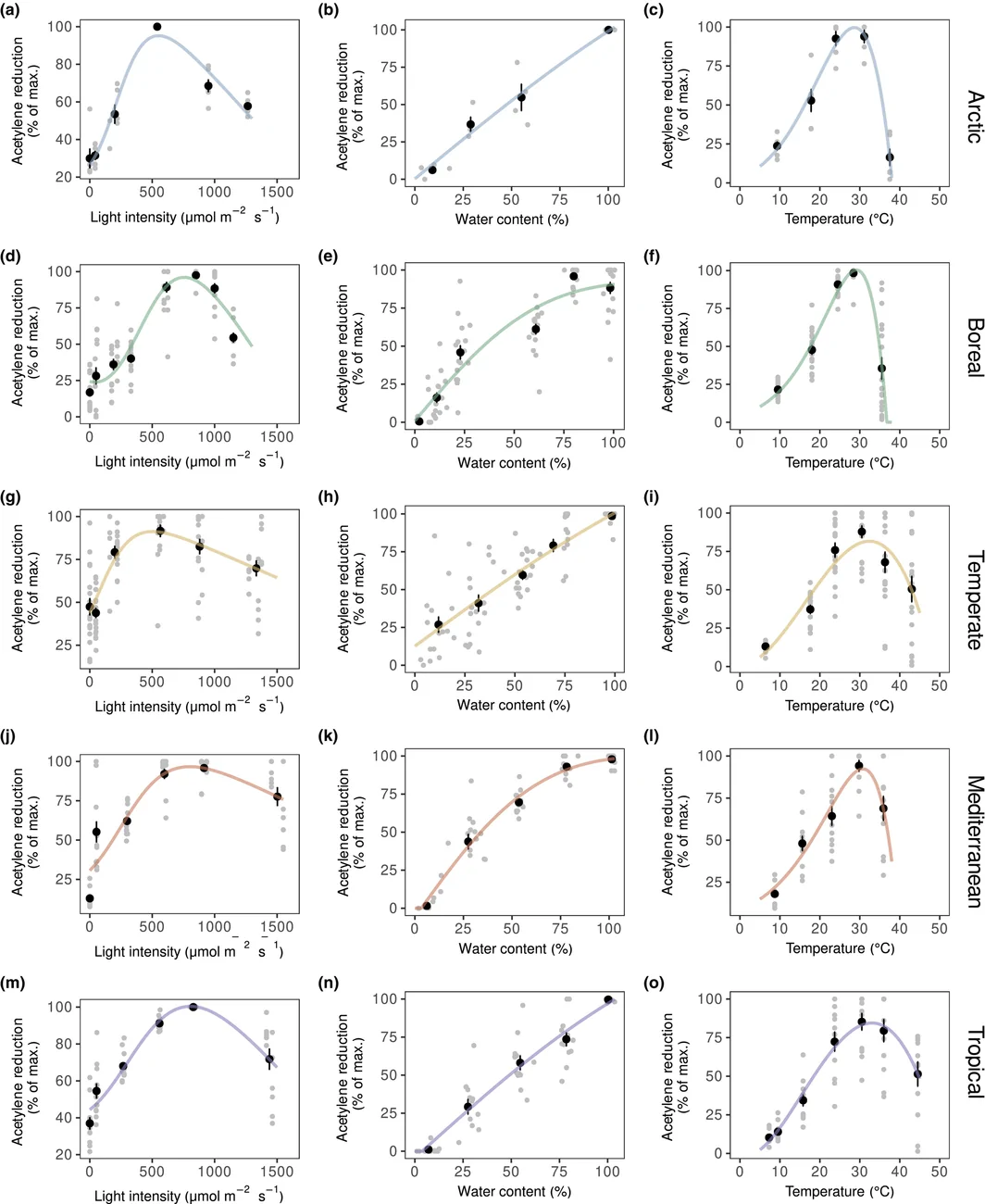
The responses of nitrogen (N) fixation to light intensity (a, d, g, j, m), relative water content (b, e, h, k, n), and temperature (c, f, i, l, o) in moss–cyanobacteria associations collected from sites within arctic (a–c), boreal (d–f), temperate (g–i), mediterranean (j–l), and tropical (m–o) ecosystems. Each data point (gray dot) represents a measured N fixation value (a total of 1068 measurements), expressed as acetylene reduction rate per moss dry mass normalized to the maximum rate of each sample (max.); black dots and error bars show ecosystem mean ± SE across all species (*n* = 6). Curves shown are maximum‐likelihood‐estimated response functions for N fixation fitted to all measurements within an ecosystem.

**Fig. 3:**
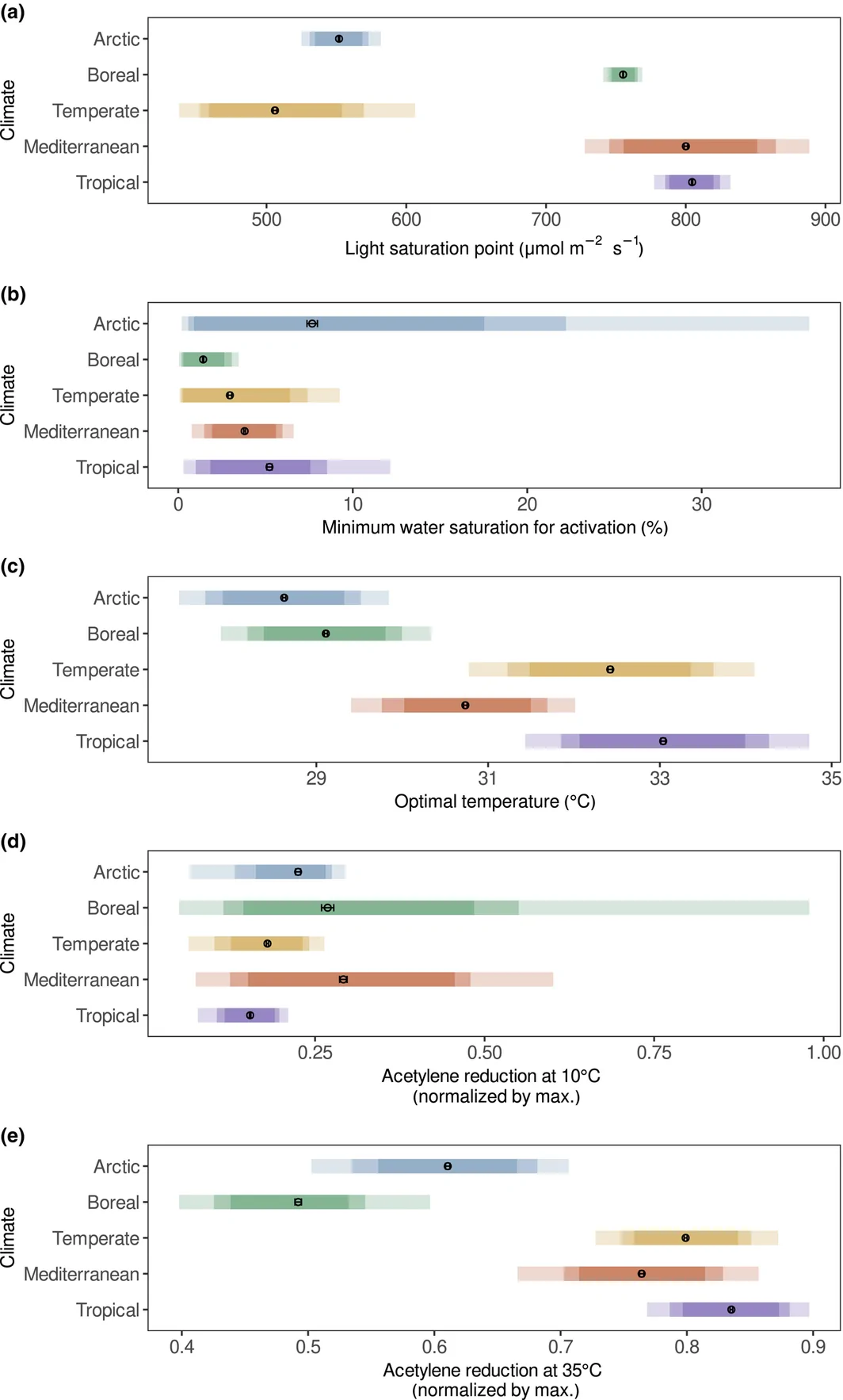
Distribution of major nitrogen (N) fixation response traits across the five sampling ecosystems with contrasting climates. (a) optimal light intensity for N fixation; (b) relative water content (RWC) at which N fixation rates reach 1% of the maximum rate, defined here as the minimum RWC for activation; (c) optimum temperature for N fixation; (d) acetylene reduction rates (as a measure of N fixation) normalized to the maximum rate under cold (10°C); and (e) warm (35°C) conditions in different sampled ecosystems. The circles indicate the mean trait values derived from the maximum‐likelihood‐estimated response functions, error bars show the SE of traits, which in some traits or ecosystems are too small to be visible. The colored bars indicate the confidence intervals at 95, 85, and 75% (the lighter colors indicate higher confidence levels). If the colored bars do not overlap with each other, the traits differ significantly from each other at the respective confidence levels.

The shape of the light‐response curves of moss‐associated N fixation was similar among the samples, with a rise to optimum light intensity followed by a drop (Fig. 2a,d,g,j,m). The optimal light intensity for N fixation ranged from 501 to 805 μmol m^−2^ s^−1^ across ecosystems with contrasting climates, showing significant variation at the 95% confidence level, with N fixation in mosses from the temperate forest and arctic tundra peaking at lower values than the other ecosystems (Fig. 3a). However, this pattern did not follow the macroclimatic gradient of light availability in the ecosystems, which, for instance, was higher in temperate than in the boreal regions (Table S1). Furthermore, the rate of decline under high light was markedly different at the 95% confidence level across ecosystems, with N fixation in mosses from the boreal and arctic declining steeper than mediterranean and temperate ecosystems (Fig. S2).

N fixation rates increased with RWC across all ecosystems with contrasting climates, though a saturation trend was only observed in boreal and mediterranean ecosystems (Fig. 2b,e,h,k,n). In addition, cyanobacteria were still active (defined as the RWC at which 1% of the maximum N fixation rate is achieved) at less than *c*. 8% RWC on average in all ecosystems (from *c*. 1% in the boreal to *c*. 8% in the arctic ecosystem). The RWC required to sustain N fixation activity did not differ significantly among ecosystems at the 95% confidence level (Fig. 3b), despite large variation in mean annual precipitation across the sampled ecosystems (Table S1).

The shape of the temperature‐response curves was also similar across ecosystems with contrasting climates, characterized by an asymmetrical pattern with a gradual increase followed by a steep decline (Fig. 2c,f,i,l,o). While the optimal temperature (Topt) for N fixation ranged from 28.1 to 33.0°C across ecosystems, Topt was significantly higher in mosses from the tropical and temperate ecosystems than in boreal and arctic ecosystems at the 95% confidence level (Fig. 3c). These differences were also reflected in N fixation activity at warm (35°C; N‐fixwarm) temperatures (Fig. 3e). At cold temperatures (10°C), the trend was not significant (95% confidence) but indicated a tendency of higher activity (N‐fixcold) in boreal and arctic ecosystems than in the warm tropical ecosystem (Fig. 3d).

### Factors driving the differences in key N fixation response physiological traits across ecosystems with different climates

**Fig. 4:**
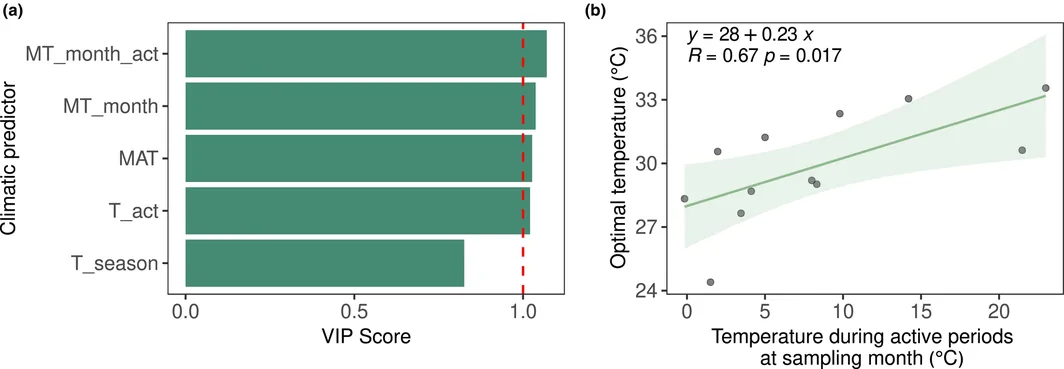
Contribution and relationship of macroclimatic variables to the optimum temperature for nitrogen (N) fixation (Topt). (a) Relative contributions of macroclimatic predictors (mean temperature during the active periods of the sampling month and the previous month (MT_month_act), mean temperature at the sampling month and the previous month (MT_month), mean annual temperature (MAT), mean temperature during active periods (T_act)), temperature seasonality (T_season), quantified by Variable importance in projection (VIP) scores, to the optimum temperature for N fixation across ecosystems with contrasting climates. The active period is defined as the moss relative water content estimated by the LiBry model is sufficient (≥ 10%) to support the N fixation activity. The vertical dashed line indicates VIP = 1, which is used as the threshold for selecting relevant variables in the model. (b) Relationship between the most important predictor, the mean temperature in the active periods during the sampling month and the previous month, and the optimum temperature of N fixation across all species and sampled ecosystems. The solid line represents the best‐fit regression model, and the shading represents the 95% confidence interval.

The optimal temperature (Topt) and N fixation activity at warm (35°C) temperatures (N‐fixwarm) differed significantly between ecosystems with different climates (Fig. 3). The contributions of annual mean temperature temperature (MAT) and annual mean temperature during the active period (T_act; defined as the period when estimated moss water content using the LiBry model is sufficient to support N fixation activity), to Topt showed similar relative importance (VIP > 1; Fig. 4a). They were also significantly linked to Topt (*P* < 0.05; Fig. S3). However, beyond the impacts of annual mean climatic variables, thermal conditions in the sampling months appeared to play at least as important a role in explaining the observed variation in Topt among ecosystems. In particular, the mean temperature during the active period of the sampling month (MT_month_act) was the strongest predictor and showed a global tendency of *c*. 0.23 increase in Topt with an increase in MT_month_act (Fig. 4b). A similar pattern was observed for N‐fixwarm, where the maximum daily mean temperature during the active period of the sampling month was the most influential variable (Fig. S4). However, in contrast to Topt, the annual mean variables showed no significant importance (Fig. S4).

**Fig. 5:**
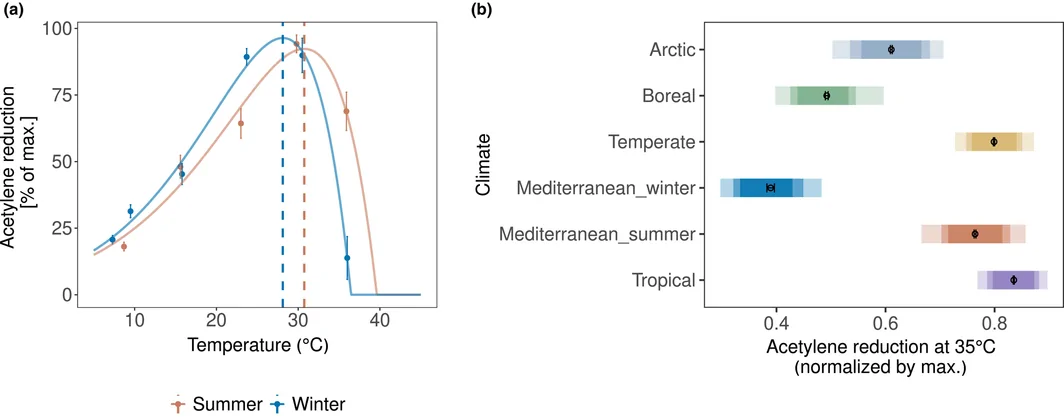
Temperature response of nitrogen (N) fixation in moss‐cyanobacteria associations. (a) Comparison of normalized N fixation rates (measured as acetylene reduction) in samples collected from Mediterranean forests during months with contrasting thermal conditions (in summer and winter). Points and error bars are mean ± SE of N fixation rate (normalized to a maximum of 100% for each sample) across two dominant species, *n* = 6. Curves shown are maximum‐likelihood‐estimated temperature response functions fitted to the data from each of two sampling periods. Vertical dashed lines are at the respective estimated optimal temperatures. (b) N fixation rate (measured as acetylene reduction; normalized to the maximum rate) at high temperature (35°C) across ecosystems with different climates. Circles indicate the mean trait values derived from the maximum‐likelihood‐estimated temperature response functions, and error bars show the SE of traits, which in some ecosystems are too small to be visible. The colored bars indicate the confidence intervals at 95, 85, and 75% (the lighter colors indicate higher confidence levels). If the bars do not overlap with each other, the traits differ significantly from each other at the respective confidence levels.

To further confirm the impact of different sampling months on temperature‐response traits, we measured the temperature‐response curves of mosses from a mediterranean forest in a contrasting period with markedly different thermal conditions (winter) compared with the first sampling conducted in summer. Mediterranean habitats exhibit pronounced intra‐annual thermal variation, with winters that are typically wet and mild, in contrast to the hot and dry conditions during the summer sampling period. Indeed, the timing of sampling with different climates substantially influenced Topt and N‐fixwarm, with significantly higher values in the summer (Topt is 30.9°C and N‐fixwarm is 0.77) than those in the winter (Topt is 28.1°C and N‐fixwarm is 0.38; see Figs 5, S5). The Topt and N‐fixwarm of moss–cyanobacteria associations sampled during the mediterranean winter were similar to, or even lower than, those observed in boreal and arctic ecosystems during spring and summer (Fig. 5b).

In addition to temperature‐response traits, the optimal light intensity for N fixation also differed significantly between ecosystems with contrasting climates. We found it was primarily shaped by the combination of annual total radiation, LAI, and maximum daily radiation, independent of whether these occur during the active period (Fig. S6).

### Nitrogen fixation efficiency across ecosystems with contrasting climates

**Fig. 6:**
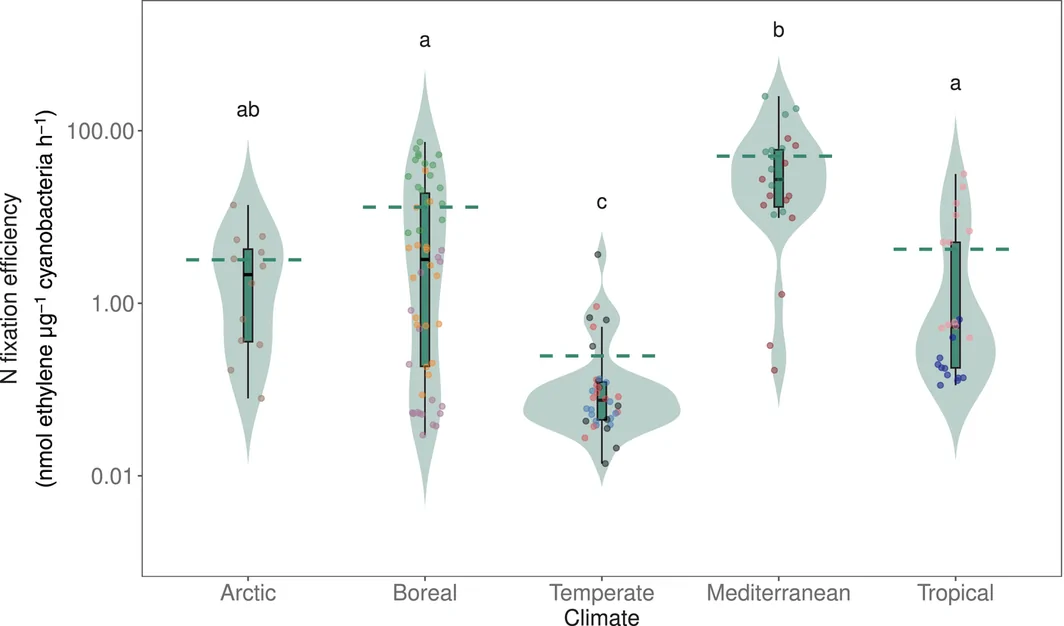
Nitrogen (N) fixation efficiency in moss–cyanobacteria associations across samples from ecosystems with different climates. Each dot represents the value for individual samples on a log‐scaled y axis, colored by species. The shape of the violins illustrates the density of the data in the samples. Boxplots within the violin plots show the median, 75^th^ percentile, and 25^th^ percentile values. Horizontal dashed lines denote the mean values of all analyzed samples within each ecosystem. Different lower‐case letters indicate significant differences between ecosystems (Dunn–Bonferroni test, *P* < 0.05).

In addition to the traits reflecting the responses of N fixation to abiotic factors, N fixation efficiency, defined as the N fixation potential per unit cyanobacterial biomass, also varied across the investigated ecosystems, with highest N fixation efficiency in the mediterranean forest, followed by the boreal, tropical, and arctic, and lowest in the temperate ecosystem (*P* < 0.001; Fig. 6). The pattern of N fixation efficiency across these ecosystems closely mirrored that of N fixation capacity (maximum acetylene reduction rate per moss biomass), despite substantial variation in cyanobacterial biomass (as indicated by phycocyanin content) across ecosystems (Fig. S7). Furthermore, cyanobacterial biomass showed no significant relationships with N fixation capacity across ecosystems, although a weak negative trend was observed in warm ecosystems (tropical and mediterranean), and a weak positive trend in the cold arctic tundra (Fig. S8).

## Discussion

Knowledge of the full range of responses of moss‐associated N fixation to key abiotic factors (light, water, temperature) and their variation across climates is currently limited, constraining our ability to estimate and predict variation in moss‐associated N fixation over the long term and across ecosystems using vegetation models. Our response curves provide direct evidence that light and temperature regulation of N fixation vary significantly across ecosystems with different climates, shaped by the local climatic conditions (partially supporting H1), while moisture limitation was consistent across ecosystems (not supporting H1).

The optimal light intensity we assessed here is higher than that experienced by moss‐cyanobacteria associations in their habitat, since it surpassed the average full radiation not intercepted by canopy cover (Table S1). These high values are consistent with previous findings that N fixation remains unsaturated at light intensities of 500 μmol m^−2^ s^−1^ in boreal and tropical ecosystems (Gundale *et al*., 2012a; Alvarenga *et al*., 2024a). Nevertheless, these values represent the light intensity measured at the moss surface. The moss‐associated N‐fixing bacteria may receive substantially lower light levels, because they are often located between overlapping moss leaves and can therefore be shaded by the moss canopy. This is consistent with previous studies showing that cyanobacteria can reach peak nitrogenase or photosynthetic activity at much lower light intensities, typically *c*. 100–200 μmol m^−2^ s^−1^ PPFD (photosynthetic photon flux density) (Jeanfils & Tack, 1992; Lennihan *et al*., 1994; Thiet *et al*., 2005). Furthermore, optimal light intensities may vary with the temperature at which light‐response curves are measured, as N fixation under high light can be higher at lower temperatures than those observed at the optimal temperature levels (*c*. 25°C; Gundale *et al*., 2012a). This light–temperature interaction may account for the observed inconsistency between the decline of N fixation rates and optimal light intensities across ecosystems with contrasting climates. Specifically, the most shade‐tolerant temperate samples exhibited a higher light tolerance than N fixation in mosses from more open arctic and boreal sites (Fig. S2). This could be due to their greater heat‐tolerance (i.e. higher N‐fixwarm) compared to arctic and boreal mosses (Fig. 3e), which allows them to persist under combined high light and high temperatures, whereas arctic and boreal mosses may experience more pronounced physiological damage (Gundale *et al*., 2012a). Additionally, the counterintuitive relationships between the optimal light intensity for N fixation and the macroclimatic light gradient in the sampled ecosystems suggest that N fixation may be more closely linked to microclimatic light conditions, similar to photosynthesis (Boehning & Burnside, 1956). Although the macroclimatic radiation variables and LAI data are coarse proxies that cannot fully represent the fine‐scale light environment experienced by mosses and associated N‐fixing bacteria, our analysis shows that the light response patterns of moss‐associated N fixation are driven by a combination of LAI and annual above‐canopy radiation (Fig. S6). This indicates that understorey light availability may help shape the optimal light intensity for N fixation across climatically contrasting ecosystems.

The response of moss‐associated N fixation to moisture, on the other hand, was similar across ecosystems with contrasting climates, which is not in accordance with H1. However, it confirms that moisture is a key limiting factor of N fixation, independent of habitat (Li *et al*., 2018; Rousk *et al*., 2018; Rousk, 2022). Regardless of the history of moisture conditions before sampling, N fixation can remain active at low moss moisture levels. Furthermore, full water saturation did not limit N fixation in any ecosystems investigated here, contrasting with the photosynthetic patterns of mosses and cyanobacteria, which typically peak at moderate RWCs (Zotz *et al*., 1997; Tamm *et al*., 2018). This divergence in optimal water content between N fixation and photosynthesis implies that, in moist tropics, N fixation may be constrained by carbon availability rather than enhanced by high moisture.

This study is the first to establish an optimal temperature range of 28–33°C for N fixation in moss–cyanobacteria associations across climates, extending the climatic constraints beyond those reported in previous work that were limited to cold‐moderate ecosystems (Jean *et al*., 2012; Gundale *et al*., 2012a, 2012b; Rousk *et al*., 2017). Compared with the differences in MAT among the cold arctic, boreal, and warm tropical ecosystems (see Table S1), the optimum temperature range across ecosystems with contrasting climates was relatively small. This narrow range suggests that the nitrogenase enzyme operates within a constrained thermal window despite large differences in ambient temperature regimes (Bytnerowicz *et al*., 2022; Deutsch *et al*., 2024). Furthermore, these numbers are substantially higher than the optimal temperature for moss net photosynthesis (generally < 25°C; Sonesson *et al*., 1992; Zotz *et al*., 2003; Colesie *et al*., 2014). However, the optimum temperature of cyanobacterial photosynthesis can be similarly high (e.g. 37°C; Tamm *et al*., 2018). This indicates that cyanobacterial N fixation may be largely supported by their own carbon assimilation, especially in warm habitats or under long‐term heat stress, rather than entirely relying on carbon supplied by the moss host (Alvarenga *et al*., 2024b). However, follow‐up experiments are needed to confirm this by for example tracing carbon between cyanobacteria and moss hosts, under different environmental conditions.

Our results reveal that both the optimum temperatures and the heat tolerance of N fixation show significant variation across ecosystems with contrasting climates, which is mostly controlled by the thermal conditions during the metabolically active period at the sampling month (Figs 4, S4). The thermal condition at the sampling month is determined by both long‐term climate as well as the timing of sampling (e.g. winter or summer). Because all samples were collected during relatively warm months, differences in sampling temperatures across ecosystems are largely driven by the long‐term climatic conditions. Thereby, the significant differences observed in temperature‐related traits across ecosystems with different climates (Fig. 3c–e) are likely driven primarily by adaptation to the temperature regimes of these ecosystems (Jean *et al*., 2012). However, a strong contrast in thermal conditions between sampling months in the mediterranean forests (i.e. early winter vs summer sampling) can exert a comparable influence on N fixation responses to temperature, with Topt increasing from 28 to 31°C (Figs 5, S5). Although the winter and summer mediterranean samples were collected from two different sites (but overlapping moss species), we found that the between‐site temperature difference was smaller than the temperature difference between winter and summer (Fig. S9). Therefore, the observed shift in Topt more likely reflects the strong contrast in thermal conditions between the two sampling periods and may have been amplified by differences between the two sites. Our results further show a tendency for lower Topt and N‐fixwarm in mediterranean compared to samples from the temperate ecosystem, despite higher annual mean temperatures (Table S1). This could be explained by our finding that the temperature responses of N fixation are shaped by the conditions when samples are biologically active (Fig. 4). Mediterranean ecosystems are typically characterized by mild, wet winters and hot, dry summers, thereby mosses are most active during early spring mornings and in the milder winter months, when temperature and moisture conditions are permissive for activity (see Fig. S10; Raggio *et al*., 2014). In other words, their active temperatures occur toward the cooler end of the local thermal gradient, whereas temperate ecosystems are generally more moist throughout the year (see Fig. S10), allowing N fixation activity across a broader temperature range (Calabria *et al*., 2020). However, because different moss species were sampled from sites across climatically contrasting ecosystems, some of the observed trait variation may be shaped by species‐specific differences in addition to environmental effects. We therefore evaluated for species effects on optimum temperature and found little evidence that this factor explained the observed variation across ecosystems strongly and significantly (see details in Notes S2).

Our study is, to the best of our knowledge, the first to assess N fixation efficiency patterns across ecosystems with different climates, revealing significant variation across these ecosystems (Fig. 6). Mediterranean mosses showed the highest N fixation efficiency, while temperate mosses exhibited the lowest (supporting H2). This pattern is likely shaped by regional climatic regimes rather than by background N deposition variation (see Table S1; Fig. S11). In the temperate rainforest, consistent and ample precipitation combined with moderate temperatures can support N fixation throughout – albeit at low rates – much of the year (see Fig. S10). By contrast, mediterranean climates, characterized by relatively low annual precipitation (Table S1) and prolonged summer droughts, constrain cyanobacteria activity to brief periods in winter and early mornings the rest of the year when temperature and moisture are favorable (Baldauf *et al*., 2021; Ma *et al*., 2023; see Fig. S10). Under these constraints, cyanobacteria in mediterranean ecosystems embody the pulse‐reserve paradigm (Garcia‐Pichel & Sala, 2022), rapidly resuscitating and activating efficient metabolic pathways such as N fixation during short favorable periods (Collins *et al*., 2008; Rousk, 2025). This strategy parallels the pre‐adaptive readiness observed in desert cyanobacteria, which for example resume photosynthesis immediately after rewetting events (Oren *et al*., 2019). Nevertheless, mosses from the boreal forest, despite being limited to a short window of activity due to extended freezing in winter and drought stress in the summer, do not exhibit such high N fixation efficiency (Fig. 6). The lower efficiency may be attributed to the low temperature during metabolically active periods (i.e. after snowmelt), which limits N fixation (Gentili *et al*., 2005). Low temperatures are also likely to select stress‐tolerant cyanobacteria that allocate more energy toward stress resistance instead of N fixation (Dasauni *et al*., 2022), thereby reducing N fixation efficiency. On the other hand, cyanobacteria in temperate rainforests likely experience reduced selective pressure for high N fixation efficiency due to the more stable conditions (i.e. moisture) over longer periods (Fig. S10). As a result, temperate mosses can maintain low but continuous N fixation activity in contrast to mediterranean mosses.

Interestingly, the relationship between cyanobacterial biomass (phycocyanin content) and N fixation capacity showed different tendencies among the investigated ecosystems (Fig. S8): in cold habitats, N fixation capacity (performed in heterocytes) and phycocyanin content (of photosynthetic cells) were positively correlated. This is consistent with the generally low N fixation efficiency (Fig. 6) that requires proportionally more energy from photosynthesis than cyanobacteria from the mediterranean site with higher N fixation efficiency. Accordingly, the trend was the opposite at the mediterranean site (see the Discussion section). At the tropical site, the correlation was also negative, likely resulting from a strong nutrient limitation on N fixation, such as phosphorus (P; Du *et al.,* 2020) and/or molybdenum (Mo; Wong *et al*., 2020). In such P‐ and Mo‐limited systems, higher photosynthetic capacity (i.e. greater phycocyanin content) might lead to exacerbated nutrient limitation of N fixation.

Even though the response curves and derived physiological response traits in our study were based on different numbers of dominant moss species collected from a few sites within each ecosystem during peak productivity periods, they provide a useful first basis for regional‐scale predictions of moss‐associated N fixation. The derived response functions may be broadly consistent among moss communities within similar climates because, first, the temperature responses of N fixation are likely constrained by broadly similar enzymatic processes across locally successful N‐fixing microorganisms occurring within a habitat (Deutsch *et al*., 2024). Second, and more convincingly, when applied to moss communities from an independent mediterranean forest site, these functions successfully simulated N fixation dynamics based on recorded light, water, and temperature (see details in Notes S3), capturing both the magnitude and temporal pattern of *in situ* rates (Fig. S12). This demonstrates that the site‐specific functions established in this study are transferable across sites.

In conclusion, our work represents a major advance in understanding moss‐associated N fixation, an increasingly recognized N input pathway that has historically been overlooked compared to legume‐associated N fixation. Specifically, our study shows how this pathway responds to key abiotic factors and how these responses vary among ecosystems spanning contrasting climates. By quantifying key physiological N fixation traits, we showed that responses of moss‐associated N fixation to light and temperature are shaped by the local climate, whereas responses to water are similar across ecosystems with contrasting climates. Our study also emphasizes the need to consider the conditions of sampling time when assessing the thermal physiology of moss‐cyanobacterial N fixation. Furthermore, the optimal light, moisture, and temperature for N fixation in moss‐cyanobacteria associations surpass the averaged conditions in their native region, suggesting a stimulation of N fixation under climate warming. However, this benefit may be offset by warming‐induced drying. Our quantified response functions of N fixation to key abiotic factors, along with N fixation efficiency across ecosystems, lay the groundwork for incorporating this process into process‐based vegetation models, although their broader applicability and use in long‐term predictions should be further evaluated with additional measurements across species, sites, and seasons. This will enable improved estimates of global N fixation patterns and better predictions of how this N input source may vary under climate change scenarios.

## Competing interests

None declared.

## Author contributions

YM and KR designed the study. YM conducted experiments, collected the data, performed data analysis, and wrote the manuscript with the support of KR.

## Disclaimer

The New Phytologist Foundation remains neutral with regard to jurisdictional claims in maps and in any institutional affiliations.

## Supporting information


**Fig. S1** Moss‐surface microclimatic vs ERA5 macroclimatic temperature.
**Fig. S2** Comparison of light‐response curves and decline slopes across ecosystems with contrasting climates.
**Fig. S3** Relationships between optimum temperature for moss‐associated nitrogen (N) fixation and macroclimatic variables.
**Fig. S4** Relative importance of macroclimatic variables for normalized nitrogen (N) fixation activity at 35°C across ecosystems with contrasting climates.
**Fig. S5** Optimum temperature across sampled ecosystems with contrasting climates and mediterranean sampling seasons.
**Fig. S6** Relative importance of macroclimatic variables for the optimal light intensity of nitrogen (N) fixation across ecosystems with contrasting climates.
**Fig. S7** Nitrogen (N) fixation capacity and phycocyanin content across ecosystems with contrasting climates.
**Fig. S8** Relationship between nitrogen (N) fixation capacity and phycocyanin content.
**Fig. S9** Comparison of mean 2 m air temperature between mediterranean sampling sites and seasons.
**Fig. S10** Simulated moss relative water content in temperate and mediterranean ecosystems.
**Fig. S11** Relationship between nitrogen (N) fixation efficiency and atmospheric N deposition.
**Fig. S12** Simulated and observed *in situ* moss‐associated nitrogenase activity at a mediterranean forest site.
**Methods S1** Variable importance in projection scores calculations.
**Notes S1** Potential effects of sample handling and repeated measurements on nitrogen fixation responses.
**Notes S2** Effects of species identity on the optimal temperature for nitrogen fixation.
**Notes S3** Application of quantified response functions in an independent mediterranean site.
**Table S1** Information on sampling sites, sampled moss species, and associated environmental data.
Please note: Wiley is not responsible for the content or functionality of any Supporting Information supplied by the authors. Any queries (other than missing material) should be directed to the *New Phytologist* Central Office.

## Data Availability

All data and R‐scripts that support the findings of this study are available in the figshare repository at doi: 10.6084/m9.figshare.31819606.

## References

[nph71472-bib-0001] Adams DG , Duggan PS . 2008. Cyanobacteria–bryophyte symbioses. Journal of Experimental Botany 59: 1047–1058. 18267939 10.1093/jxb/ern005

[nph71472-bib-0002] Alvarenga DO , Clasen LA , Thomsen AMR , Andersen RF , Rousk K . 2024a. Light drives nitrogen fixation in tropical montane cloud forests in Costa Rica. Science of the Total Environment 940: 173631. 38823705 10.1016/j.scitotenv.2024.173631

[nph71472-bib-0003] Alvarenga DO , Priemé A , Rousk K . 2024b. The feather moss *Hylocomium splendens* affects the transcriptional profile of a symbiotic cyanobacterium in relation to acquisition and turnover of key nutrients. Microbial Ecology 87: 49. 38427046 10.1007/s00248-024-02363-6 PMC10907420

[nph71472-bib-0004] Arróniz‐Crespo M , Pérez‐Ortega S , los RAD , Green TGA , Ochoa‐Hueso R , Casermeiro MÁ , de la CMT , Pintado A , Palacios D , Rozzi R *et al*. 2014. Bryophyte‐cyanobacteria associations during primary succession in recently deglaciated areas of Tierra del Fuego (Chile). PLoS ONE 9: e96081. 24819926 10.1371/journal.pone.0096081 PMC4018330

[nph71472-bib-0005] Baldauf S , Porada P , Raggio J , Maestre FT , Tietjen B . 2021. Relative humidity predominantly determines long‐term biocrust‐forming lichen cover in drylands under climate change. Journal of Ecology 109: 1370–1385.

[nph71472-bib-0006] Boehning RH , Burnside CA . 1956. The effect of light intensity on rate of apparent photosynthesis in leaves of sun and shade plants. American Journal of Botany 43: 557–561.

[nph71472-bib-0007] Bolker B , R Development Core Team . 2023. bbmle: tools for general maximum likelihood estimation . R package v.1.0.25.1. [WWW document] URL https://CRAN.R-project.org/package=bbmle.

[nph71472-bib-0008] Bolker BM . 2008. Ecological models and data in R. Princeton, NJ, USA: Princeton University Press.

[nph71472-bib-0009] Burnham KP , Anderson DR . 2004. Multimodel inference: understanding AIC and BIC in model selection. Sociological Methods & Research 33: 261–304.

[nph71472-bib-0010] Bytnerowicz T , Akana P , Griffin K , Menge D . 2022. Temperature sensitivity of woody nitrogen fixation across species and growing temperatures. Nature Plants 8: 209–216. 35115725 10.1038/s41477-021-01090-x

[nph71472-bib-0011] Calabria LM , Petersen KS , Bidwell A , Hamman ST . 2020. Moss‐cyanobacteria associations as a novel source of biological N_2_‐fixation in temperate grasslands. Plant and Soil 456: 307–321.

[nph71472-bib-0012] Chong I‐G , Jun C‐H . 2005. Performance of some variable selection methods when multicollinearity is present. Chemometrics and Intelligent Laboratory Systems 78: 103–112.

[nph71472-bib-0013] Colesie C , Allan Green TG , Haferkamp I , Büdel B . 2014. Habitat stress initiates changes in composition, CO_2_ gas exchange and C‐allocation as life traits in biological soil crusts. The ISME Journal 8: 2104–2115. 24694713 10.1038/ismej.2014.47 PMC4184013

[nph71472-bib-0014] Collins SL , Sinsabaugh RL , Crenshaw C , Green L , Porras‐Alfaro A , Stursova M , Zeglin LH . 2008. Pulse dynamics and microbial processes in aridland ecosystems. Journal of Ecology 96: 413–420.

[nph71472-bib-0015] Dasauni K , Divya , Nailwal TK . 2022. Cyanobacteria in cold ecosystem: tolerance and adaptation. In: Goel R , Soni R , Suyal DC , Khan M , eds. Survival strategies in cold‐adapted microorganisms. Singapore: Springer, 1–29.

[nph71472-bib-0016] DeLuca TH , Zackrisson O , Gentili F , Sellstedt A , Nilsson M‐C . 2007. Ecosystem controls on nitrogen fixation in boreal feather moss communities. Oecologia 152: 121–130. 17219131 10.1007/s00442-006-0626-6

[nph71472-bib-0017] DeLuca TH , Zackrisson O , Nilsson M‐C , Sellstedt A . 2002. Quantifying nitrogen‐fixation in feather moss carpets of boreal forests. Nature 419: 917–920. 12410308 10.1038/nature01051

[nph71472-bib-0018] Deutsch C , Inomura K , Luo Y‐W , Wang Y‐P . 2024. Projecting global biological N_2_ fixation under climate warming across land and ocean. Trends in Microbiology 32: 546–553. 38262802 10.1016/j.tim.2023.12.007

[nph71472-bib-0080] Du E , Terrer C , Pellegrini AFA , Ahlström A , van Lissa CJ , Zhao X , Xia N , Wu X , Jackson RB . 2020. Global patterns of terrestrial nitrogen and phosphorus limitation. Nature Geoscience 13: 221–226.

[nph71472-bib-0019] Elbert W , Weber B , Burrows S , Steinkamp J , Büdel B , Andreae MO , Pöschl U . 2012. Contribution of cryptogamic covers to the global cycles of carbon and nitrogen. Nature Geoscience 5: 459–462.

[nph71472-bib-0020] Fan X , Yuan G , Liu W . 2022. Response strategies of N‐fixation by epiphytic bryophytes to water change in a subtropical montane cloud forest. Ecological Indicators 135: 108527.

[nph71472-bib-0021] Garcia‐Pichel F , Sala O . 2022. Expanding the pulse–reserve paradigm to microorganisms on the basis of differential reserve management strategies. Bioscience 72: 638–650.

[nph71472-bib-0022] Gentili F , Nilsson M‐C , Zackrisson O , DeLuca TH , Sellstedt A . 2005. Physiological and molecular diversity of feather moss associative N_2_‐fixing cyanobacteria. Journal of Experimental Botany 56: 3121–3127. 16263908 10.1093/jxb/eri309

[nph71472-bib-0023] Gundale MJ , Nilsson M , Bansal S , Jäderlund A . 2012a. The interactive effects of temperature and light on biological nitrogen fixation in boreal forests. New Phytologist 194: 453–463. 22329746 10.1111/j.1469-8137.2012.04071.x

[nph71472-bib-0024] Gundale MJ , Wardle DA , Nilsson M‐C . 2012b. The effect of altered macroclimate on N‐fixation by boreal feather mosses. Biology Letters 8: 805–808. 22696285 10.1098/rsbl.2012.0429 PMC3440997

[nph71472-bib-0025] Hájek T , Tuittila E‐S , Ilomets M , Laiho R . 2009. Light responses of mire mosses – a key to survival after water‐level drawdown? Oikos 118: 240–250.

[nph71472-bib-0027] Holland‐Moritz H , Stuart JEM , Lewis LR , Miller SN , Mack MC , Ponciano JM , McDaniel SF , Fierer N . 2021. The bacterial communities of Alaskan mosses and their contributions to N2‐fixation. Microbiome 9: 53. 33622403 10.1186/s40168-021-01001-4 PMC7903681

[nph71472-bib-0028] Houlton BZ , Wang Y‐P , Vitousek PM , Field CB . 2008. A unifying framework for dinitrogen fixation in the terrestrial biosphere. Nature 454: 327–330. 18563086 10.1038/nature07028

[nph71472-bib-0029] Jean M‐E , Cassar N , Setzer C , Bellenger J‐P . 2012. Short‐term N_2_ fixation kinetics in a moss‐associated cyanobacteria. Environmental Science & Technology 46: 8667–8671. 22849538 10.1021/es3018539

[nph71472-bib-0030] Jeanfils J , Tack JP . 1992. Identification and study of growth and nitrogenase activity of nitrogen fixing cyanobacteria from tropical soil. Vegetatio 103: 59–66.

[nph71472-bib-0031] Kassambara A . 2023. rstatix: pipe‐friendly framework for basic statistical tests . R package v.0.7.2. [WWW document] URL https://CRAN.R-project.org/package=rstatix.

[nph71472-bib-0032] Kou D , Yang G , Li F , Feng X , Zhang D , Mao C , Zhang Q , Peng Y , Ji C , Zhu Q *et al*. 2020. Progressive nitrogen limitation across the Tibetan alpine permafrost region. Nature Communications 11: 3331. 10.1038/s41467-020-17169-6 PMC7335038 32620773

[nph71472-bib-0033] Lange OL , Büdel B , Heber U , Meyer A , Zellner H , Green TGA . 1993. Temperate rainforest lichens in New Zealand: high thallus water content can severely limit photosynthetic CO_2_ exchange. Oecologia 95: 303–313. 28314003 10.1007/BF00320981

[nph71472-bib-0034] Lange OL , Tenhunen JD . 1981. Moisture content and CO_2_ exchange of lichens. II. Depression of net photosynthesis in *Ramalina maciformis* at high water content is caused by increased thallus carbon dioxide diffusion resistance. Oecologia 51: 426–429. 28310031 10.1007/BF00540917

[nph71472-bib-0035] Lennihan R , Chapin DM , Dickson LG . 1994. Nitrogen fixation and photosynthesis in high arctic forms of Nostoc commune. Canadian Journal of Botany 72: 940–945.

[nph71472-bib-0036] Levinsen JUG , Yuan M , Michelsen A , Rousk K . 2025. Is moss‐associated nitrogen fixation controlled by the same factors across shoots, species and sites? Environmental and Experimental Botany 239: 106262. 41244956 10.1016/j.envexpbot.2025.106262 PMC12614486

[nph71472-bib-0037] Li D , Zhang Q , Xiao K , Wang Z , Wang K . 2018. Divergent responses of biological nitrogen fixation in soil, litter and moss to temperature and moisture in a karst forest, southwest China. Soil Biology and Biochemistry 118: 1–7.

[nph71472-bib-0038] Liland KH , Mevik B‐H , Wehrens R . 2024. pls: partial least squares and principal component regression . R package v.2.8-5. [WWW document] URL https://CRAN.R-project.org/package=pls.

[nph71472-bib-0039] Lindo Z , Nilsson M‐C , Gundale MJ . 2013. Bryophyte‐cyanobacteria associations as regulators of the northern latitude carbon balance in response to global change. Global Change Biology 19: 2022–2035. 23505142 10.1111/gcb.12175

[nph71472-bib-0040] Ma Y , Weber B , Kratz A , Raggio J , Colesie C , Veste M , Bader MY , Porada P . 2023. Exploring environmental and physiological drivers of the annual carbon budget of biocrusts from various climatic zones with a mechanistic data‐driven model. Biogeosciences 20: 2553–2572.

[nph71472-bib-0041] McCulloch LA , Porder S . 2020. Lower nodule biomass with increased nitrogenase efficiency in *Robinia pseudoacacia* seedlings when grown under low soil phosphorus conditions. SN Applied Sciences 2: 1785.

[nph71472-bib-0042] Mehmood T , Liland KH , Snipen L , Sæbø S . 2012. A review of variable selection methods in partial least squares regression. Chemometrics and Intelligent Laboratory Systems 118: 62–69.

[nph71472-bib-0043] Nikolić N , Zotz G , Bader MY . 2024. Modelling the carbon balance in bryophytes and lichens: presentation of PoiCarb 1.0, a new model for explaining distribution patterns and predicting climate‐change effects. American Journal of Botany 111: e16266. 38038342 10.1002/ajb2.16266

[nph71472-bib-0045] Oren N , Raanan H , Kedem I , Turjeman A , Bronstein M , Kaplan A , Murik O . 2019. Desert cyanobacteria prepare in advance for dehydration and rewetting: the role of light and temperature sensing. Molecular Ecology 28: 2305–2320. 31025457 10.1111/mec.15074

[nph71472-bib-0046] Permin A , Horwath AB , Metcalfe DB , Priemé A , Rousk K . 2022. High nitrogen‐fixing rates associated with ground‐covering mosses in a tropical mountain cloud forest will decrease drastically in a future climate. Functional Ecology 36: 1772–1781.

[nph71472-bib-0047] Porada P , Van Stan JT , Kleidon A . 2018. Significant contribution of non‐vascular vegetation to global rainfall interception. Nature Geoscience 11: 563–567.

[nph71472-bib-0048] Porada P , Weber B , Elbert W , Pöschl U , Kleidon A . 2013. Estimating global carbon uptake by lichens and bryophytes with a process‐based model. Biogeosciences 10: 6989–7033.

[nph71472-bib-0049] Puzorjov A , McCormick AJ . 2020. Phycobiliproteins from extreme environments and their potential applications. Journal of Experimental Botany 71: 3827–3842. 32188986 10.1093/jxb/eraa139

[nph71472-bib-0050] R Core Team . 2023. R: a language and environment for statistical computing. Vienna, Austria: R Foundation for Statistical Computing.

[nph71472-bib-0051] Raggio J , Pintado A , Vivas M , Sancho LG , Büdel B , Colesie C , Weber B , Schroeter B , Lázaro R , Green TGA . 2014. Continuous chlorophyll fluorescence, gas exchange and microclimate monitoring in a natural soil crust habitat in *Tabernas badlands*, Almería, Spain: progressing towards a model to understand productivity. Biodiversity and Conservation 23: 1809–1826.

[nph71472-bib-0052] Ramm E , Liu C , Ambus P , Butterbach‐Bahl K , Hu B , Martikainen PJ , Marushchak ME , Mueller CW , Rennenberg H , Schloter M *et al*. 2022. A review of the importance of mineral nitrogen cycling in the plant‐soil‐microbe system of permafrost‐affected soils—changing the paradigm. Environmental Research Letters 17: 013004.

[nph71472-bib-0054] Reich PB , Hobbie SE , Lee T , Ellsworth DS , West JB , Tilman D , Knops JMH , Naeem S , Trost J . 2006. Nitrogen limitation constrains sustainability of ecosystem response to CO_2_ . Nature 440: 922–925. 16612381 10.1038/nature04486

[nph71472-bib-0055] Renaudin M , Blasi C , Bradley RL , Bellenger J‐P . 2022. New insights into the drivers of moss‐associated nitrogen fixation and cyanobacterial biomass in the eastern Canadian boreal forest. Journal of Ecology 110: 1403–1418.

[nph71472-bib-0056] Renaudin M , Darnajoux R , Bellenger J‐P . 2021. Quantification of moss‐associated cyanobacteria using phycocyanin pigment extraction. Frontiers in Microbiology 11: 611792. 33469453 10.3389/fmicb.2020.611792 PMC7813775

[nph71472-bib-0057] Ribet J , Drevon J‐J . 1996. The phosphorus requirement of N_2_‐fixing and urea‐fed *Acacia mangium* . New Phytologist 132: 383–390. 26763634 10.1111/j.1469-8137.1996.tb01858.x

[nph71472-bib-0058] Rousk K . 2025. Moss‐cyanobacteria associations: a model for studying symbiotic interactions and evolutionary strategies. American Journal of Botany 112: e70086. 40794385 10.1002/ajb2.70086 PMC12374560

[nph71472-bib-0059] Rousk K . 2022. Biotic and abiotic controls of nitrogen fixation in cyanobacteria–moss associations. New Phytologist 235: 1330–1335. 35687087 10.1111/nph.18264

[nph71472-bib-0060] Rousk K , Pedersen PA , Dyrnum K , Michelsen A . 2017. The interactive effects of temperature and moisture on nitrogen fixation in two temperate‐arctic mosses. Theoretical and Experimental Plant Physiology 29: 25–36.

[nph71472-bib-0061] Rousk K , Sorensen PL , Lett S , Michelsen A . 2015. Across‐habitat comparison of diazotroph activity in the subarctic. Microbial Ecology 69: 778–787. 25403111 10.1007/s00248-014-0534-y

[nph71472-bib-0062] Rousk K , Sorensen PL , Michelsen A . 2018. What drives biological nitrogen fixation in high arctic tundra: moisture or temperature? Ecosphere 9: e02117.

[nph71472-bib-0063] Rousk K , Villarreal AJC . 2025. Time to end the vascular plant chauvinism. Nature Plants 11: 3. 39695272 10.1038/s41477-024-01876-9

[nph71472-bib-0064] Schöllhorn R , Burris RH . 1967. Acetylene as a competitive inhibitor of N_2_ fixation. Proceedings of the National Academy of Sciences, USA 58: 213–216. 10.1073/pnas.58.1.213 PMC335619 5231601

[nph71472-bib-0065] Slate ML , Antoninka A , Bailey L , Berdugo MB , Callaghan DA , Cárdenas M , Chmielewski MW , Fenton NJ , Holland‐Moritz H , Hopkins S *et al*. 2024. Impact of changing climate on bryophyte contributions to terrestrial water, carbon, and nitrogen cycles. New Phytologist 242: 2411–2429. 38659154 10.1111/nph.19772

[nph71472-bib-0066] Sonesson M , Gehrke C , Tjus M . 1992. CO_2_ environment, microclimate and photosynthetic characteristics of the moss *Hylocomium splendens* in a subarctic habitat. Oecologia 92: 23–29. 28311808 10.1007/BF00317258

[nph71472-bib-0067] Stewart KJ , Lamb EG , Coxson DS , Siciliano SD . 2011. Bryophyte‐cyanobacterial associations as a key factor in N_2_‐fixation across the Canadian Arctic. Plant and Soil 344: 335–346.

[nph71472-bib-0068] Tamm A , Caesar J , Kunz N , Colesie C , Reichenberger H , Weber B . 2018. Ecophysiological properties of three biological soil crust types and their photoautotrophs from the Succulent Karoo, South Africa. Plant and Soil 429: 127–146.

[nph71472-bib-0069] Thiet RK , Boerner REJ , Nagy M , Jardine R . 2005. The effect of biological soil crusts on throughput of rainwater and N into lake Michigan sand dune soils. Plant and Soil 278: 235–251.

[nph71472-bib-0070] Venables WN , Ripley BD . 2002. Modern applied statistics with S. New York, NY, USA: Springer.

[nph71472-bib-0071] Vitousek PM , Cassman K , Cleveland C , Crews T , Field CB , Grimm NB , Howarth RW , Marino R , Martinelli L , Rastetter EB *et al*. 2002. Towards an ecological understanding of biological nitrogen fixation. In: Boyer EW , Howarth RW , eds. The nitrogen cycle at regional to global scales. Dordrecht, the Netherlands: Springer Netherlands, 1–45.

[nph71472-bib-0072] Wang Y‐P , Houlton BZ . 2009. Nitrogen constraints on terrestrial carbon uptake: implications for the global carbon‐climate feedback. Geophysical Research Letters 36: L24403.

[nph71472-bib-0073] Wickham H . 2016. ggplot2: elegant graphics for data analysis. New York, NY, USA: Springer‐Verlag.

[nph71472-bib-0074] Wong MY , Mahowald NM , Marino R , Williams ER , Chellam S , Howarth RW . 2020. Natural atmospheric deposition of molybdenum: a global model and implications for tropical forests. Biogeochemistry 149: 159–174.

[nph71472-bib-0075] Yan W , Hunt LA . 1999. An equation for modelling the temperature response of plants using only the cardinal temperatures. Annals of Botany 84: 607–614.

[nph71472-bib-0076] Zhou W , Bai Y , Xie Y , Wei B , Wanek W , Rousk K , Noyce G , Zhang D , Peng Y , Yang Y . 2026. Key role of moss in supplementing nitrogen for plant growth under warming in a permafrost ecosystem. Proceedings of the National Academy of Sciences, USA 123: e2516443123. 10.1073/pnas.2516443123 PMC12933092 41706876

[nph71472-bib-0077] Zielke M , Ekker AS , Olsen RA , Spjelkavik S , Solheim B . 2002. The influence of abiotic factors on biological nitrogen fixation in different types of vegetation in the high arctic, Svalbard. Arctic, Antarctic, and Alpine Research 34: 293–299.

[nph71472-bib-0078] Zotz G , Büdel B , Meyer A , Zellner H , Lange OL . 1997. Water relations and CO_2_ exchange of tropical bryophytes in a lower montane rain forest in Panama. Botanica Acta 110: 9–17.

[nph71472-bib-0079] Zotz G , Schultz S , Rottenberger S . 2003. Are tropical lowlands a marginal habitat for macrolichens? Evidence from a field study with *Parmotrema endosulphureum* in Panama. Flora ‐ Morphology, Distribution, Functional Ecology of Plants 198: 71–77.

