# Supplementary material for: Nonuniform temperature and light relationships of moss‐associated nitrogen fixation across climates
Source: New Phytol. 2026 Aug 3;252(1):276–91. doi: 10.1111/nph.71472 (PMC13539968; doi:10.1111/nph.71472)
Supplement: Supplementary file 1 [file NPH-252-276-s001.docx]

## *New Phytologist* Supporting Information

Article title: Nonuniform temperature and light relationships of moss-associated nitrogen fixation across climates

Authors: Yunyao Ma, Kathrin Rousk

Article acceptance date: 8 July 2026

The following Supporting Information is available for this article:

**Fig. S1** Moss-surface microclimatic versus ERA5 macroclimatic temperature.

**Fig. S2** Comparison of light response curves and decline slopes across ecosystems with contrasting climates.

**Fig. S3** Relationships between optimum temperature for moss-associated nitrogen (N) fixation and macroclimatic variables.

**Fig. S4** Relative importance of macroclimatic variables for normalized nitrogen (N) fixation activity at 35 °C across ecosystems with contrasting climates.

**Fig. S5** Optimum temperature across sampled ecosystems with contrasting climates and mediterranean sampling seasons.

**Fig. S6** Relative importance of macroclimatic variables for the optimal light intensity of nitrogen (N) fixation across ecosystems with contrasting climates.

**Fig. S7** Nitrogen (N) fixation capacity and phycocyanin content across ecosystems with contrasting climates.

**Fig. S8** Relationship between nitrogen (N) fixation capacity and phycocyanin content.

**Fig. S9** Comparison of mean 2 m air temperature between mediterranean sampling sites and seasons.

**Fig. S10** Simulated moss relative water content in temperate and mediterranean ecosystems.

**Fig. S11** Relationship between nitrogen (N) fixation efficiency and atmospheric N deposition.

**Fig. S12** Simulated and observed *in situ* moss-associated nitrogenase activity at a mediterranean forest site.

**Table S1** Information on sampling sites, sampled moss species, and associated environmental data.

**Methods S1** Variable importance in projection (VIP) scores calculations

**Notes S1** Potential effects of sample handling and repeated measurements on nitrogen fixation responses

**Notes S2** Effects of species identity on the optimal temperature for nitrogen fixation

**Notes S3** Application of quantified response functions in an independent mediterranean site

**Fig. S1** Moss-surface microclimatic versus ERA5 macroclimatic temperature. Moss-surface microclimatic temperature at a mediterranean forest site in Sardinia, Italy, compared with macroclimatic temperature extracted from the ERA5 dataset at the coordinates of the sampling site. The dashed line represents the 1:1 relationship, whereas the blue solid line represents the best-fit linear regression to data points and the shaded area indicates the 95% confidence interval.

**Fig. S2** Comparison of light response curves and decline slopes across ecosystems with contrasting climates. (a) Fitted light response curves for nitrogen (N) fixation measured as acetylene reduction. The curves of N fixation (normalized to a maximum of 100% for each sample) in response to light in moss-cyanobacteria associations collected from sites within five ecosystems with different climates (arctic, boreal, temperate, mediterranean, and tropical) are fitted with equation 1 in the main text using maximum-likelihood estimation approach. (b) modelled decline slopes across different ecosystems with contrasting climates. The circles indicate the mean values of the maximum slope in the declining phase of maximum-likelihood-estimated light response curves across ecosystems. Larger slope values indicate a more gradual decline in light response after saturation, suggesting lower sensitivity to excess light. The coloured bars indicate the confidence intervals at 95%, 85%, and 75% (the lighter colours indicate higher confidence levels). If the bars do not overlap with each other, it means that the traits differ significantly from each other at the respective confidence levels.

**Fig. S3** Relationships between optimum temperature for moss-associated nitrogen (N) fixation and macroclimatic variables. Optimum temperature for N fixation associated with all moss species from different macroclimates was plotted against (a) annual mean temperature and (b) mean temperature during the active period, when the moss relative water content estimated by the LiBry model is sufficient (≥10%) to support the N fixation activity. Solid lines represent the best-fit regression models, and shading areas represent the 95% confidence interval.

**Fig. S4** Relative importance of macroclimatic variables for normalized nitrogen (N) fixation activity at 35 °C across ecosystems with contrasting climates. The variables include maximum daily mean temperature during active period in the month of sampling and the previous month (MaxT_month_act), maximum daily mean temperature in the sampling and the previous month (MaxT_month), temperature seasonality (calculated as the standard deviation of monthly mean temperature), maximum annual temperature during active period (Tmax_act), maximum annual temperature (Tmax), as well as temperature seasonality (T_season). Active period is defined as the moss relative water content estimated by the LiBry model is sufficient (≥10%) to support the N fixation activity. The relative importance is quantified by VIP scores using the equation S1. The vertical dashed line indicates VIP = 1, which is used as the threshold for selecting relevant variables in the model.

**Fig. S5** Optimum temperature across sampled ecosystems with contrasting climates and mediterranean sampling seasons. Optimum temperature was compared across ecosystems with different climates and between two sampling months with markedly contrasting thermal conditions at the mediterranean site (summer and winter month). The circles indicate the mean trait values derived from the maximum-likelihood-estimated temperature response functions, and the coloured bars indicate the confidence intervals at 95%, 85%, and 75% (the lighter colours indicate higher confidence levels). If the bars do not overlap with each other, the traits differ significantly from each other at the respective confidence levels.

**Fig. S6** Relative importance of macroclimatic variables for the optimal light intensity of nitrogen (N) fixation across ecosystems with contrasting climates. The variables include annual total radiation (sum_rad_total), maximum daily total radiation (rad_max), maximum daily total radiation during active period (rad_max_act), leaf area index (LAI), annual total radiation during active period (sum_rad_total_act), average daily hours with radiation > 100 W m^-2^ (sunshine), and total radiation in the sampling month (sum_rad_month). The active period is defined as the time when the moss relative water content is sufficient (≥ 10%) to support the N fixation activity. The relative importance is quantified by Variable importance in projection (VIP) scores using the equation S1. The vertical dashed line indicates VIP = 1, which is used as the threshold for selecting relevant variables in the model.

**Fig. S7** Nitrogen (N) fixation capacity and phycocyanin content across ecosystems with contrasting climates. N fixation capacity is defined as the moss-associated N fixation rates (measured as acetylene reduction) under optimal light, moisture, and temperature conditions. Shown are means ± SE; tropical and mediterranean, n=24; temperate, n = 36; boreal, n = 54; arctic, n = 12. Different lowercase letters indicate significant differences between ecosystems (Dunn-Bonferroni test for N fixation capacity and Tukey HSD test for phycocyanin content, *p* < 0.05).

**Fig. S8** Relationship between nitrogen (N) fixation capacity and phycocyanin content. N fixation capacity (nmol g dw^-1^ h^-1^) under optimal light, moisture, and temperature was plotted against cyanobacterial biomass, quantified by phycocyanin content, across moss species and sampled ecosystems (Arctic, Boreal, Temperate, Mediterranean, and Tropical). Colored points and lines represent the species-specific measured values and linear fits, respectively. Black dashed lines show the overall fit for each ecosystem across all species in that ecosystem. Shading indicates 95% confidence intervals.

**Fig. S9** Comparison of mean 2 m air temperature between mediterranean sampling sites and seasons. Mean 2 m air temperature was extracted from the ERA5 dataset for 2000–2019, averaged annually for summer (June–August) and winter (November–February). Temperature differences between the two mediterranean sampling sites (France and Italy; Δsite) and seasonal temperature differences within each site (Δseason) are shown. France represents the winter sampling site for temperature response measurements, whereas Italy represents the summer sampling site.

**Fig. S10** Simulated moss relative water content in temperate and mediterranean ecosystems. Daily (a, f) and diurnal (b-e; g-j) patterns of simulated relative water content in moss-cyanobacteria associations from the temperate (a-e) and mediterranean (f-j) ecosystems by the LiBry model. Active nitrogen fixation is defined as occurring when the relative water content > 0.1, as indicated by the red threshold dashed line. Panels (b) to (e) or (g) to (j) represent the patterns of average hourly relative water content from January to March (JFM), April to June (AMJ), July to September (JAS), and October to December (OND), respectively.

**Fig. S11** Relationship between nitrogen (N) fixation efficiency and atmospheric N deposition. N fixation efficiency was quantified for moss samples, while annual atmospheric N deposition rates were extracted from Lamarque *et al*. (2013) for the corresponding sampling sites across five climatically contrasting ecosystems: Arctic, Boreal, Temperate, Mediterranean, and Tropical. The solid line represents the best-fit regression model, and the shading represents the 95% confidence interval.

**Fig. S12** Simulated and observed *in situ* moss-associated nitrogenase activity at a mediterranean forest site. Simulated acetylene reduction rates (nitrogenase activity; Simulation; orange line) were compared with observed *in situ* values (Measurement; green line) for 10 moss community samples from a forest (F) site in Sardinia, Italy. Each panel shows the comparison for a single sample (replicates 1-10 at the forest (F) site) at four time points (night, early morning, noon, and afternoon) over 5 days (13-17 October, 2025). Points in green and orange represent the observed nitrogenase activity and simulated values at each time point, respectively. The simulated values were modelled based on observed temperature, light intensity, and water content using the quantified response functions derived in this study.

**Table S1** Information on sampling sites, sampled moss species, and associated environmental data. The table includes the climate zone, region, geographic coordinates, sampled moss species, and key environmental variables for each site, including mean annual temperature (MAT), mean annual precipitation, mean annual short-wave radiation, and mean annual nitrogen deposition. Climatic variables were extracted from the ERA5 global climate database and reflect ecosystem-specific site averages over the period 2009-2019, whereas nitrogen deposition rates were extracted from Lamarque et al. (2013) and represent averages for 2005-2014.

| Climate zone | Region | Latitude  (° N) | Longitude  (° E) | Sampled species | MAT (°C) | Mean annual precipitation (mm) | Mean annual short-wave radiation (μmol m⁻² s⁻¹) | Annual nitrogen deposition (kg N ha^-1^ yr^-1^) |
| --- | --- | --- | --- | --- | --- | --- | --- | --- |
| Arctic | Iceland | 63.47 | -19.25 | *Racomitrium lanuginosum* | 4.7 | 1901 | 198.4 | 4.69 |
|  |  | 63.44 | -20.26 |  |  |  |  |  |
|  |  | 63.96 | -16.81 |  |  |  |  |  |
|  |  | 63.80 | -19.15 |  |  |  |  |  |
|  |  | 64.39 | -21.25 |  |  |  |  |  |
|  |  | 63.75 | -18.16 |  |  |  |  |  |
| Boreal | North Sweden | 66.02 | 19.85 | [*Hylocomium splendens, Pleurozium schreberi*](https://www.britishbryologicalsociety.org.uk/learning/species-finder/ptilium-crista-castrensis/) | 1.9 | 437 | 197.3 | 2.63 |
|  |  | 64.98 | 19.57 | [*Ptilium crista-castrensis*](https://www.britishbryologicalsociety.org.uk/learning/species-finder/ptilium-crista-castrensis/) |  |  |  |  |
| Temperate | New Zealand | -44.93 | 167.95 | *Dicranoloma robustum, Ptychomnion aciculare* | 8.7 | 4333 | 229.8 | 0.92 |
|  |  | -42.90 | 171.18 | *Leucobryum javense* |  |  |  |  |
| Mediterranean | Italy | 42.36 | 10.89 | *Leucodon sciuroides, Hypnum cupressiforme* | 17.7 | 667 | 396.6 | 6.94 |
| Tropical | Borneo | 5.62 | 117.18 | *Pogonatum cirratum, Hypnodendron diversifolium* | 23.4 | 3183 | 433.5 | 2.86 |

**Methods S1** Variable importance in projection (VIP) scores calculations

The relative importance of different macroclimatic predictors in the partial least squares (PLS) regression model for the key physiological traits across ecosystems with contrasting climates was quantified using the following equation:

${VIP}_{j}=\sqrt{p\sum_{1}^{A} \left( \frac{W_{ja}^{2}S_{a}^{2}}{\sum_{1}^{A} S_{a}^{2}} \right)}$	(S1)

where p is the number of predictors, A is the total number of PLS components, W_ja_ is the weight of predictor j in component a, S_a_^2^ is the explained variance of the response variable by component a.

**Notes S1** Potential effects of sample handling and repeated measurements on nitrogen fixation responses

*S1.1 Effects of storage and acclimation on manipulation experiments*

After sampling, the samples were stored at 4 °C in darkness until measurements in September 2024. Although storage duration differed among mosses from different ecosystems (see Materials and Methods), we do not expect this to have substantially affected nitrogen (N) fixation. Samples were stored under conditions that closely reflected their field moisture status at the time of collection: Samples from the boreal and mediterranean forests were very dry; therefore, these mosses and their associated microbes were likely dormant or only minimally active during storage. Storage of less than 3 months and subsequent rehydration after storage were found to not substantially affect moss-associated N fixation activity compared with freshly collected samples (Hagge *et al.*, 2025). In contrast, samples from the arctic tundra, tropical and temperate forests were moist, but their metabolic activity during storage at 4°C and dark was expected to be very low (Puzorjov & McCormick, 2020). Therefore, these storage conditions likely had only minimal effects on bacterial diversity and composition (Brandt *et al.*, 2014; Choo *et al.*, 2015). Based on our temperature- and light-response curves, we extrapolated N fixation rates for samples from each ecosystem to 4°C, darkness, and full water saturation. These extrapolated rates remained very low, at 0.28, 0.04, and 0.11 nmol g^-1^dw h^-1^ for arctic, temperate, and tropical samples, respectively, less than 1.6% for arctic, temperate, and 0.2% of the maximum activity for tropical samples. Therefore, we consider that storage at 4°C and darkness did not substantially affect the observed patterns of moss-associated N fixation.

Before experiments, samples were kept in a growth chamber for one week to reactivate after storage. The acclimation conditions were chosen to represent the common conditions that all samples could reasonably experience in the field (12:12 h light-dark cycle, a light intensity of 150 µmol m^−2^ s ^−1^, a temperature of 20 °C in the light and 17 °C in the dark, and relative humidity of 80%). Therefore, we do not expect this short acclimation period to have driven substantial changes in moss-associated microbial communities and physiological states relative to their original *in situ* conditions. This is also supported by our results, as we still observed clear and significant differences among ecosystems in the light and temperature response curves. If the acclimation period had largely homogenized the moss–microbe associations, we would have expected stronger convergence in response curves and physiological traits among ecosystems.

To further examine whether prolonged storage duration and the acclimation period may have shifted N fixation activity and physiology away from field conditions, we compared the first measurements from the light response assays (at 50 µmol photons m⁻² s⁻¹, 25°C, and full water saturation) with additional pre-storage baseline measurements available for the temperate samples. For the temperate samples, pre-storage N fixation activity was measured immediately after field sampling, before storage and acclimation. These measurements were conducted at 13 µmol m⁻² s⁻¹, 18 °C, and full water saturation. To account for the incubation temperature difference between the pre-storage measurements and the first N fixation measurements, we corrected the pre-storage rates to 25 °C using the fitted temperature response curve for the temperate samples. Specifically, the measured values were divided by the temperature-response scaling factor derived from the fitted temperature response function for temperate samples. We then compared these temperature-corrected pre-storage values with the first measurement point after storage. N fixation activity was not significantly different between those two measurements (Wilcoxon rank-sum test, *p* > 0.05; see Supporting Information Fig. A), although the pre-storage values tended to be slightly higher. This suggests that the longest storage duration under cool and dark conditions and the acclimation period for temperate samples did not substantially alter N fixation activity.

Together, these results suggest that storage and *ex situ* standardization of the samples did not appear to cause a clear shift in initial N fixation activity relative to their original field state.

*S1.2 Effects of repeated sample use on manipulation experiments*

Because nitrogenase activity was measured repeatedly on the same samples, potential time-dependent changes in sample physiology and carry-over effects from previous measurement conditions cannot be excluded and may have influenced the quantified response curves. However, the same procedure was applied consistently across all moss samples, making the comparisons among ecosystems comparable. Furthermore, several lines of evidence suggest that our measurements, especially those conducted at later stages of the assays, still reasonably represent the physiological state of the samples.

First, we performed an additional test on mediterranean samples by measuring the water response curve in the reverse direction, from high to low water content. The comparison with response curves measured in the forward direction (Supporting Information Fig. B) suggests that time-dependent or carry-over effects played only a minor role in affecting N fixation activity, because the original forward measurement approach did not inhibit N fixation rates at later measurement stages (at high water saturation levels). The quantified trait, namely the minimum water saturation required for activation, did not differ substantially between the two measurement approaches.

While we did not conduct additional light and temperature response measurements backwards, we further evaluated whether repeated sample use affected the observed light- and temperature-response patterns by comparing N fixation rates for the same moss species reported in other studies with those measured at our study sites. Rousk et al. (2017) measured N fixation rates of *Pleurozium schreberi* from Abisko, Sweden without repeated sample use and reported higher activity at 30 °C than at 20 °C, with rates of 4.78 and 2.66 nmol g⁻¹ dw h⁻¹, respectively, corresponding to a 1.8-fold difference. A consistent pattern was observed in our boreal data for the same species under full saturation: although the measurement at approximately 30 °C (28.4 °C) was conducted later in the temperature-response sequence, N fixation remained higher than at approximately 20 °C (18 °C), with rates of 0.46 and 0.24 nmol g⁻¹ dw h⁻¹, respectively. This 1.92-fold difference is comparable to that observed in the Abisko samples. Furthermore, another study (Alvarenga *et al.*, 2026) measured N fixation rates of *Hylocomium splendens* and *Pleurozium schreberi* from Abisko one day after exposure to optimal temperature (25°C), full water saturation, and light conditions of 200 ± 25 μmol photons m⁻² s⁻¹, without repeated use of the samples. The measured values, 14.23 ± 1.8 nmol g^-1^ dw h^-1^ for *Hylocomium splendens* and 10.62 ± 2.05 nmol g^-1^ dw h^-1^ for *Pleurozium schreberi*, were lower than or comparable to the values measured in our light response experiment at a light level of around 200 μmol photons m⁻² s⁻¹ (46.73 nmol g^-1^ dw h^-1^ for *H. splendens* and 10.75 nmol g^-1^ dw h^-1^ for *P. schreber*i). Overall, these comparisons suggest that measurements obtained at later stages of the light and temperature response experiments in this study were not strongly depressed relative to the expected response patterns.

Together, the response patterns were not strongly affected by repeated measurements or accumulated treatment effects.

**Fig. A** Effects of storage on moss-associated nitrogen (N) fixation in temperate samples. Comparison of N fixation rates between measurements conducted immediately after field sampling (Fresh field) and the first measurement in the light response assay after seven months of storage and acclimation periods (After storage). Each dot represents an individual measurement of the N fixation rate for each sample, expressed as ethylene production per moss dry mass and incubation time. Boxplots show the median, 75th percentile, and 25th percentile values. ns indicates no significant difference (Wilcoxon rank-sum test; *p* > 0.05).

**Fig. B** Water response of nitrogen (N) fixation in moss–cyanobacteria associations during moistening and drying. (a) Comparison of water response curves for mediterranean samples during moistening (orange; Water uptake) and drying (green; Water loss). Small, semi-transparent dots represent individual measurements of N fixation, expressed as acetylene reduction rates per moss dry mass and normalized to the maximum rate of each sample. Larger points and error bars represent the ecosystem mean ± SE across two dominant moss species (n = 6). The curves represent water response functions fitted by maximum likelihood to data from the two hydration manipulations. (b) Minimum water saturation required to activate nitrogen fixation, defined as the water saturation at which N fixation rates reached 1% of the maximum rate. Circles indicate mean trait values derived from the maximum-likelihood-estimated water response functions measured during moistening (Water uptake) or drying (Water loss), and error bars show the standard errors of the trait estimates. Coloured horizontal bars indicate 95%, 85% and 75% confidence intervals, with lighter colours representing higher confidence levels. Overlapping intervals suggest that trait estimates do not differ significantly at the corresponding confidence level.

**Notes S2** Effects of species identity on the optimal temperature for nitrogen fixation

We further evaluated the contribution of species identity to variation in the derived optimal temperature of N fixation (Topt) across ecosystems with contrasting climates using fixed-effect model comparisons. To this end, we first fitted a baseline linear model with mean monthly active temperature as the only explanatory variable (“lm” function from the R package “stats”). Mean monthly active temperature was selected because it was identified as the most important environmental driver of Topt variation across ecosystems (Fig. 4a). We then fitted an additional linear model that included species identity as an additional predictor. This model was compared with the baseline model using ANOVA (R package “stats”) to test whether the inclusion of species identity significantly improved model fit. The results showed that including species identity as an additional predictor did not significantly improve model fit compared with the baseline model containing mean monthly active temperature alone (*p* = 0.55). This suggests that species identity played only a minor role in explaining variation in Topt beyond mean monthly active temperature. All these analyses were performed with R software version 4.2.3 (R Core Team, 2023).

**Notes S3** Application of quantified response functions in an independent mediterranean site

Field measurements of *in situ* N fixation were conducted from October 13 to 17, 2025 in Sardinia, Italy (39°20’56.6” N, 8°37’33.7” E). N fixation was measured in a forest plot. At this plot, 10 moss communities dominated by common species (e.g. *Leucodon sciuroides*, *Leptodon smithii*) were sampled. N fixation rates were measured four times per day to capture diel variation, including early morning, midday, afternoon, and nighttime. Microclimatic conditions were continuously monitored using temperature loggers (iButton; iButtonLink LLC) and light sensors (HOBO Micro Station; Onset Computer Corporation).

For each measurement, moss samples were placed into 50-ml transparent vials, and their fresh mass was recorded prior to incubation to estimate their water content. Nitrogen fixation was quantified using the acetylene reduction assay (ARA). Briefly, 5 ml of acetylene was injected into each gas-tight vial, and 6 ml gas samples were collected after a 2-hour incubation period. Gas samples were stored in sealed 6ml vials until analysis. Following field incubations, all moss samples were harvested and transported to the University of Copenhagen, where potential maximum N fixation rates were determined using the ARA under optimal laboratory conditions (550 µmol m⁻² s⁻¹ light, fully saturated water content, and ~28 °C). Gas samples were analysed using gas chromatography to quantify ethylene production as a proxy for nitrogenase activity.

Subsequently, we simulated N fixation rates for each time point by applying the quantified response functions in this study to the measured maximum N fixation rates in laboratory and monitored field light, temperature, and water content using the following equation. The simulated values relatively matched the measured *in situ* rates well in both temporal pattern and magnitude (Supporting Information Fig. S12).

$simulate={max}_{rate}*f_{T}\left( T,a,b,Topt,ymax \right)*f_{W}\left( WC,a,b,c \right)*f_{L}\left( Light,a,b,c,ymax \right)$

where max_rate_ represents the maximum N fixation rate of each sample measured under optimal laboratory conditions (see above). f_T_, f_W_, and f_L_ represent the quantified temperature, water, and light response functions of N fixation, respectively (equations 1-3). We applied parameters calibrated using data from the mediterranean site, specifically temperature-response parameters fitted to winter conditions (see Supporting Information Table A and B), consistent with our field observations.

**Table A** Response function parameters and estimated traits across ecosystems with contrasting climates. Parameters for the light, water, and temperature response of nitrogen fixation (assessed with acetylene reduction assay) associated with mosses and estimated traits across five climatically contrasting ecosystems. Parameters are for equations 1-3 in the main text. These values and equations form the curves seen in Fig. 2 and Supporting Information Fig. S2, while estimated traits are implemented in Fig. 3.

|  | | Arctic | Boreal | Temperate | Mediterranean | Tropical |
| --- | --- | --- | --- | --- | --- | --- |
| Light response | a (95% CI) | 0.0073 (0.0064, 0.0082) | 0.0053 (0.005, 0.0057) | 0.0071 (0.0053, 0.0089) | 0.0049 (0.004, 0.006) | 0.0039 (0.0035, 0.0042) |
|  | b (95% CI) | 205.23 (205.228, 205.234) | 399.4515 (399.4511, 399.4519) | 61.20029 (61.20022, 61.20035) | 249.0631 (249.0631, 249.0632) | 300.1112 (300.1111, 300.1112) |
|  | c (95% CI) | 7.3e-4 (6.2e-4, 8.45e-4) | 1.39e-3 (1.34e-3, 1.45e-3) | 3.15e-4 (2.77e-4, 3.54e-4) | 3.87e-4 (3.34e-4, 4.45e-4) | 7.83e-4 (7.46e-4, 8.23e-4) |
|  | Optimal light (μmol m^-2^ s^-1^) (95% CI) | 550.9 (525.1, 581.8) | 755.6 (741.1, 768.7) | 500.8 (437.5, 606.3) | 800.6 (727.7, 888.4) | 805.2 (777.4, 832.0) |
| Water response | a (95% CI) | 3.68 (-4.73, 11.71) | 1.46 (1.18, 1.71) | 3.10 (-1.08, 7.08) | 1.57 (1.42, 1.72) | 2.99 (0.21, 5.62) |
|  | b (95% CI) | 3.45 (-5.96, 12.35) | 0.94 (0.54, 1.30) | 3.20 (-2.11, 8.22) | 0.88 (0.67, 1.07) | 2.62 (-0.50, 5.57) |
|  | c (95% CI) | 0.003 (-0.138, 0.131) | 0.009 (-0.052, 0.068) | 0.126 (0.024, 0.221) | -0.059 (-0.123, 0.004) | -0.043 (-0.136, 0.047) |
|  | Minimum water saturation for activation (%) (95% CI) | 7.7 (0.2, 36) | 1.4 (0.05, 3.5) | 3.0 (0.08, 9.3) | 3.8 (0.77, 6.6) | 5.2 (0.3, 12.2) |
| Temperature response | a (95% CI) | 38.13 (37.74, 38.50) | 36.76 (36.25, 37.29) | 48.56 (45.93, 51.27) | 39.41 (38.22, 40.67) | 50.71 (47.95, 53.51) |
|  | b (95% CI) | -17.82 (-51.87, 17.90) | -58.03 (-222.59, 114.68) | -0.85 (-11.20, 9.92) | -252.187 (-252.188, -252.186) | 2.86 (-3.22, 8.54) |
|  | Optimal temperature (Topt; °C) (95% CI) | 28.65 (27.4, 29.85) | 29.13 (27.89, 30.34) | 32.41 (30.78, 34.10) | 30.86 (30.20, 31.53) | 33.03 (31.43, 34.74) |
|  | Normalized Acetylene reduction at 10 °C (95% CI) | 0.24 (0.066, 0.29) | 0.21 (0.050, 0.98) | 0.19 (0.06, 0.26) | 0.25 (0.19, 0.31) | 0.17 (0.08, 0.21) |
|  | Normalized Acetylene reduction at 35 °C (95% CI) | 0.62 (0.50, 0.71) | 0.49 (0.40, 0.60) | 0.80 (0.73, 0.87) | 0.77 (0.70, 0.85) | 0.84 (0.77, 0.90) |

**Table B** Parameters for the temperature response function of nitrogen fixation and estimated traits for mosses collected in the mediterranean winter. Parameters are for equation 3 in the main text. These values and equations form the curves seen in Fig. 5a, while estimated traits are implemented in Fig. 5b and Supporting Information Fig. S5.

| Parameter/trait | Mean value (95% CI) |
| --- | --- |
| a | 36.47 (36.16, 36.76) |
| b | -88.75 (-322.92, 157.03) |
| Optimal temperature (Topt; °C) | 28.12 (26.88, 29.34) |
| Normalized Acetylene reduction at 10 °C | 0.29 (0.12, 0.89) |
| Normalized Acetylene reduction at 35 °C | 0.38 (0.30, 0.48) |

**References**

**Alvarenga DO, Wynns JT, Nesme J, Priemé A, Rousk K**. **2026**. Climate Change Impacts the Structure and Nitrogen-Fixing Activities of Subarctic Feather Moss Microbiomes Across a Precipitation Gradient. *Global Change Biology* **32**: e70718.

**Brandt FB, Breidenbach B, Brenzinger K, Conrad R**. **2014**. Impact of short-term storage temperature on determination of microbial community composition and abundance in aerated forest soil and anoxic pond sediment samples. *Systematic and Applied Microbiology* **37**: 570–577.

**Choo JM, Leong LE, Rogers GB**. **2015**. Sample storage conditions significantly influence faecal microbiome profiles. *Scientific Reports* **5**: 16350.

**Hagge P, King-Doonan E, Lutzoni F, Miadlikowska J, Aguero B, Whitby H, Umbanhowar C, Colesie C, Arnold AE, Yitong E, *et al.*** **2025**. Nitrogen fixation in Arctic lichens and mosses: A survey across circumpolar subzones. *Science of The Total Environment* **999**: 180264.

**Lamarque J-F, Dentener F, McConnell J, Ro C-U, Shaw M, Vet R, Bergmann D, Cameron-Smith P, Dalsoren S, Doherty R, *et al.*** **2013**. Multi-model mean nitrogen and sulfur deposition from the Atmospheric Chemistry and Climate Model Intercomparison Project (ACCMIP): evaluation of historical and projected future changes. *Atmospheric Chemistry and Physics* **13**: 7997–8018.

**Puzorjov A, McCormick AJ**. **2020**. Phycobiliproteins from extreme environments and their potential applications. *Journal of Experimental Botany* **71**: 3827–3842.

**R Core Team**. **2023**. *R: A Language and Environment for Statistical Computing*. Vienna, Austria: R Foundation for Statistical Computing.

**Rousk K, Pedersen PA, Dyrnum K, Michelsen A**. **2017**. The interactive effects of temperature and moisture on nitrogen fixation in two temperate-arctic mosses. *Theoretical and Experimental Plant Physiology* **29**: 25–36.
